# Recent Advancements in TiO_2_ Nanostructures: Sustainable Synthesis and Gas Sensing

**DOI:** 10.3390/nano13081424

**Published:** 2023-04-20

**Authors:** Gayan W. C. Kumarage, Hadjer Hakkoum, Elisabetta Comini

**Affiliations:** Sensor Lab, Department of Information Engineering, University of Brescia, Via Valotti 9, 25133 Brescia, Italy; g.wadumasthree@unibs.it (G.W.C.K.); h.hakkoum@unibs.it (H.H.)

**Keywords:** TiO_2_, nanostructures, low-dimensional, chemical sensors, gas sensor

## Abstract

The search for sustainable technology-driven advancements in material synthesis is a new norm, which ensures a low impact on the environment, production cost, and workers’ health. In this context, non-toxic, non-hazardous, and low-cost materials and their synthesis methods are integrated to compete with existing physical and chemical methods. From this perspective, titanium oxide (TiO_2_) is one of the fascinating materials because of its non-toxicity, biocompatibility, and potential of growing by sustainable methods. Accordingly, TiO_2_ is extensively used in gas-sensing devices. Yet, many TiO_2_ nanostructures are still synthesized with a lack of mindfulness of environmental impact and sustainable methods, which results in a serious burden on practical commercialization. This review provides a general outline of the advantages and disadvantages of conventional and sustainable methods of TiO_2_ preparation. Additionally, a detailed discussion on sustainable growth methods for green synthesis is included. Furthermore, gas-sensing applications and approaches to improve the key functionality of sensors, including response time, recovery time, repeatability, and stability, are discussed in detail in the latter parts of the review. At the end, a concluding discussion is included to provide guidelines for the selection of sustainable synthesis methods and techniques to improve the gas-sensing properties of TiO_2_.

## 1. Introduction

**Figure 1:**
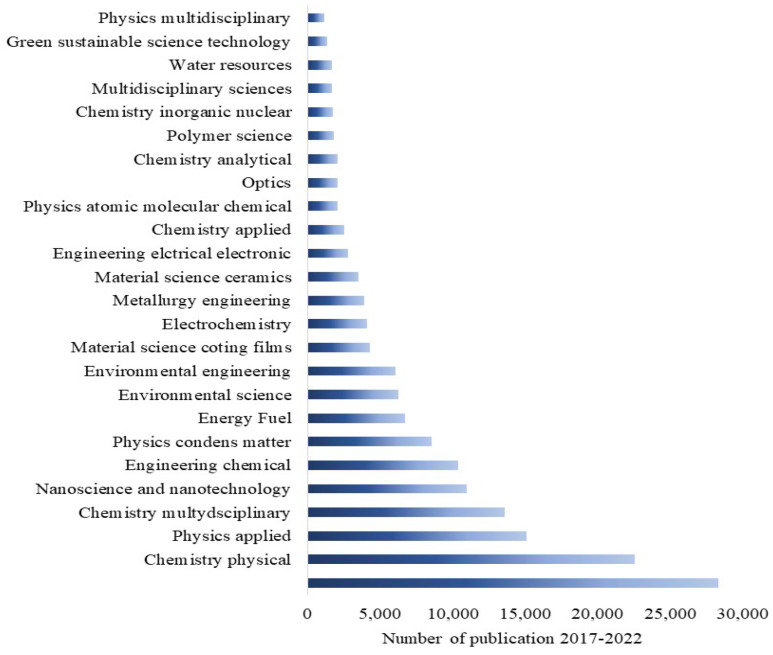
The widespread publications on TiO_2_ in various research fields in the last five years. Resource: Web of Science.

Having a happy life is a basic need for all living beings, but this is still a challenge due to aging, which speeds up health issues, resulting in death at the end [1]. Additionally, many factors, such as health pandemics, poor food quality, and poor environmental conditions, significantly affect living conditions. However, these issues can be managed through advances in medicine and diagnostic systems to control disease outbreaks and the use of affordable, portable instruments to ensure food quality. One way to help combat the environmental pollution crisis is by utilizing smart and portable monitoring systems, such as chemical sensors. Hence, there is a strong demand for innovative ideas that incorporate novel concepts in physics and chemistry in smart material processing and device integration. Metal oxides (MOXs) have the potential to be an excellent and economical choice as a raw material in this context [2,3,4]. Titanium dioxide (TiO_2_), specifically among MOXs, possesses distinctive characteristics that make it appropriate for use in these areas, as shown by its widespread use in research, particularly in the field of materials science over the past five years (Figure 1).

**Figure 2:**
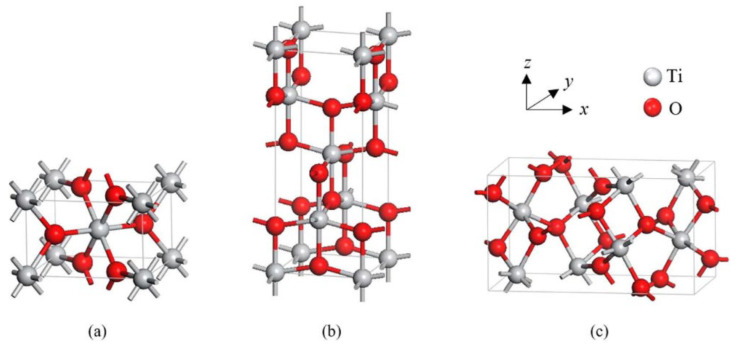
Tetragonal structures of crystalline forms of (**a**) rutile and (**b**) anatase, and orthorhombic structure of (**c**) brookite TiO_2_. Reprinted from [6].

TiO_2_ is primarily found in three different crystal structures: rutile, anatase, and brookite. Both anatase and brookite have a tetragonal form, while brookite has an orthorhombic structure. The illustration of their unit cells is shown in Figure 2. These two metastable phases are known for their ability to irreversibly transform into the stable rutile phase at temperatures between 400 and 800 °C, based on various factors [5]. The two most frequently used forms of TiO_2_ in gas-sensing applications are tetragonal anatase (I41/amd) and rutile (P42/mnm). The lattice measurements for anatase are a = b = 0.376 nm and c = 0.948 nm, while rutile’s lattice measurements are a = b = 0.459 nm and c = 0.296 nm. In both anatase and rutile, each titanium atom is bonded to six oxygen atoms, creating a TiO_6_ octahedron.

**Figure 3:**
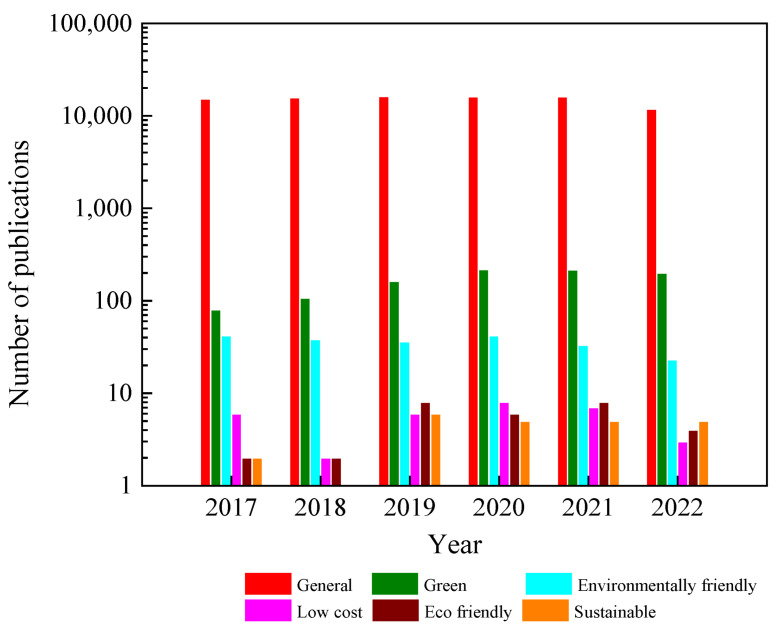
Synthesis of TiO_2_ by growth methods in the last five years. Resource: Web of Science.

Currently, TiO_2_ is extensively synthesized in a variety of morphological forms, including nanoparticles, nanowires, nanobelts, nanotubes, nanorods, and nanosheets, for various applications. However, conventional methods of preparation are still used, as depicted in Figure 3, leaving the question of sustainable synthesis open. Thus, the first section of this review focuses on informing researchers about sustainable methods for growing TiO_2_ using “green”, “low-cost”, “environmentally friendly”, and “eco-friendly” techniques.

Returning to the earlier discussion about a “happy life”, having a clean and fresh environment is crucial. Without it, exposure to pollutants (chemicals/gases) may lead to health problems, such as headaches; respiratory illness; dizziness; fatigue; eye, nose, and skin irritation; and potentially even death. Hence, it is essential to monitor and measure toxic and exhaust gases for the well-being of society, energy conservation, and environmental preservation. Chemical gas sensors are utilized to monitor chemicals/gases in both industrial and household settings. [7]. Gas sensors are devices used to detect and measure the presence of specific gases (chemicals) in an environment. These sensors play a crucial role in many industries and applications, including air quality monitoring, industrial process control, and safety systems. Gas sensors come in a variety of types, each with unique capabilities and advantages [8]. Conductometric gas sensors are a type of gas-sensing technology that utilizes electrical conductivity to detect specific gases. Compared to other types of gas sensors, conductometric gas sensors have several advantages, such as being portable, small, affordable, and dependable, along with a low power consumption. These sensors work by measuring changes in conductivity that occur in response to the presence of specific gases. In order to enhance their selectivity and sensitivity, as well as to ensure fast response and recovery times, ongoing efforts are underway to find more suitable materials for these sensors [9].

**Figure 4:**
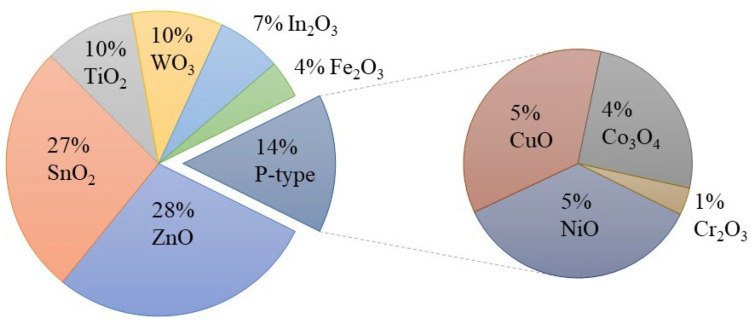
Use of metal oxides in gas-sensing applications from 2017 to 2022. Source: Web of Science.

**Figure 5:**
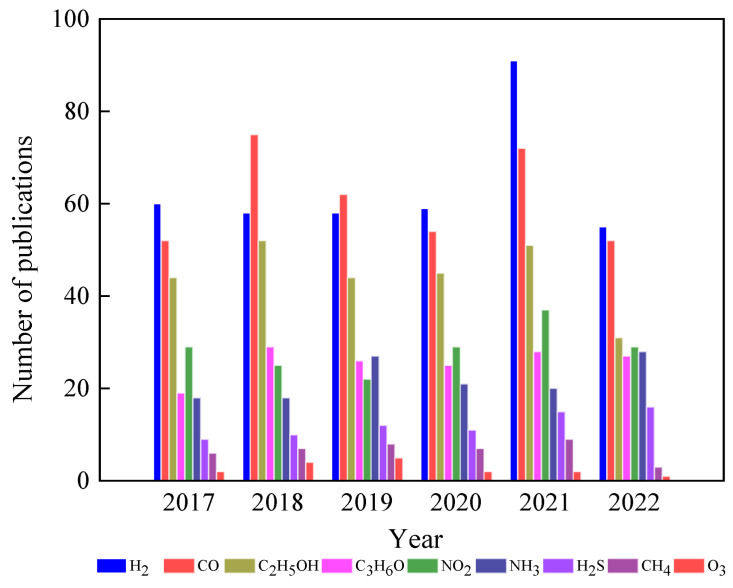
Use of TiO_2_ in gas-sensing applications in the last five years. Source: Web of Science.

In this field, MOX gas sensors are gaining significant attention as they possess many desired properties for an ideal sensor. As shown in Figure 4, there have been numerous efforts to utilize different MOXs in gas-sensing applications. Among these MOXs, TiO_2_ has been extensively studied for various gas-sensing applications, including hydrogen (H_2_), carbon monoxide (CO), ethanol (C_2_H_5_OH), acetone (C_3_H_6_O), and ozone (O_3_), in recent years (Figure 5) [10,11,12]. This article will delve into the advances made in TiO_2_ for gas-sensing applications and provide a comprehensive evaluation of its gas-sensing properties. Additionally, the gas-sensing mechanisms and prospects for sustainable growth and gas-sensing applications of TiO_2_ will also be discussed at the end of the article.

## 2. Gas-Sensing Mechanisms

A chemical sensor has two main functions: a receptor function and a transducer function. In the receptor function, the analyte compound interacts with the surface of MOXs. Meanwhile, the transducer mechanism changes a chemical signal into an electrical one in conductometric sensors. There are two well-established sensing mechanisms for MOX (TiO_2_)-based gas sensors; these include the (i) oxygen-vacancy mechanism and (ii) ionosorption mechanism [13]. The first mechanism operates based on the surface reduction/oxidation of MOXs and the concurrent alteration of surface oxygen vacancies in response to exposure to reducing and oxidizing gases [14,15]. In essence, the first step in the process involves the interaction of an analyte gas with the MOX surface, causing the removal of lattice oxygen from the surface. This creates a positive-ion vacancy, freeing electrons that enter the conduction band (CB) and reducing electrical resistance in n-type MOXs. When the gas is no longer present, oxygen fills the vacancy and takes electrons from the CB, increasing electrical resistance. Although this model is quite intricate, some details are not fully documented.

The ionosorption mechanism is widely recognized as the predominant model. This model relies on the modulation of the adsorption and desorption of oxygen species (O^−^ and O^2−^) on the MOX surface. When exposed to atmospheric air, oxygen molecules are adsorbed onto the MOX surface through two methods: physisorption and chemisorption. Physisorption is driven by van der Waals forces, with no charge transfer occurring between MOXs and oxygen. In chemisorption, oxygen molecules are tightly bound to MOXs due to the transfer of charge between MOXs and oxygen molecules. This makes chemisorption the most crucial step in this gas-sensing mechanism.

**Figure 6:**
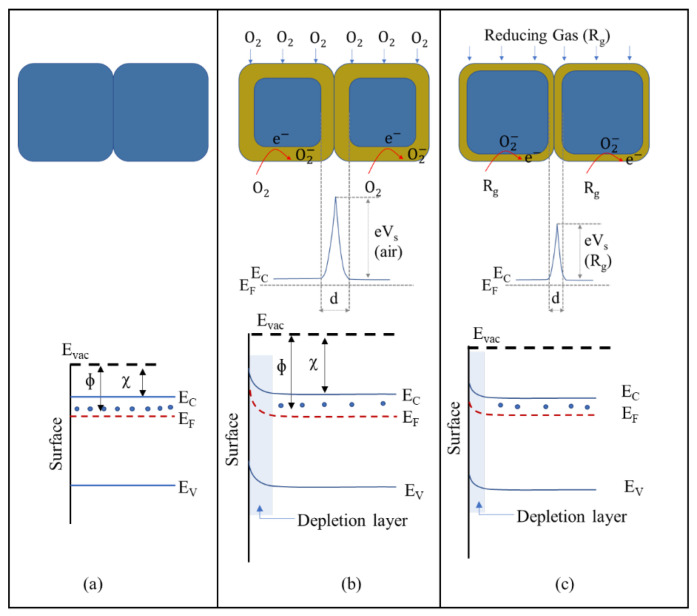
Energy-band diagram of a typical n-type MOX semiconductor: (**a**) flat band, (**b**) when an electron-depletion layer is formed due to chemisorption of oxygen molecules, and (**c**) when a reducing gas interacts with oxygen species.

In this process, oxygen molecules interact with the conduction-band electrons of MOXs and generate oxygen species on the MOX surface. The type of oxygen species formed depends on the operating temperature of the sensors. For example, O^−^ is formed when the temperature is between 150 and 400 °C, while O^2−^ is formed when the temperature is higher than 400 °C [16,17]. When conduction-band electrons are captured by oxygen molecules, a depleted electron region forms on the MOX surface. This leads to an upward bend of the energy band in n-type MOXs, such TiO_2_ (Figure 6a,b), thus reducing conductance. The presence of reducing gases (e.g., H_2_, CO, and CH_4_) interacts with oxygen species, thereby adding electrons to the conduction band and decreasing the depletion-layer width, causing a downward bend of the energy band and increasing conductance (Figure 6c). Conversely, conductance decreases in the presence of oxidizing gases (e.g., H_2_S and NO_2_). However, for complex molecules (C_2_H_5_OH and C_3_H_6_O), the working principle is not as straightforward.

## 3. Sustainable Synthesis Methods of TiO_2_ Nanostructures

**Figure 7:**
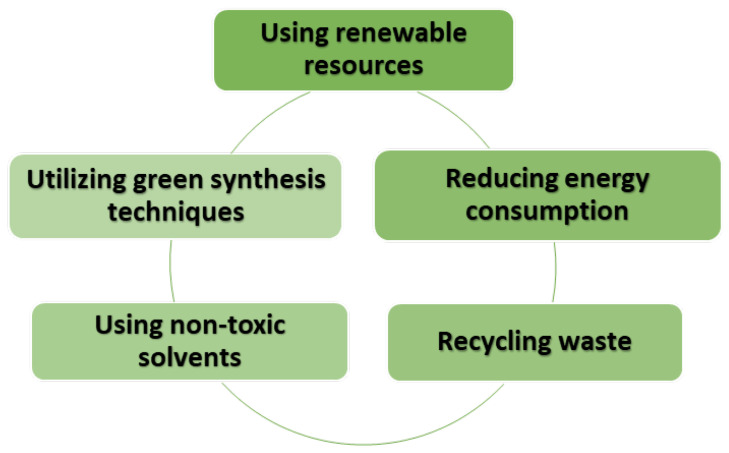
Eco-sustainable synthesis of TiO_2_: steps for a greener future.

In recent years, there has been a notable surge in the efforts made by researchers to create TiO_2_ through greener and more sustainable processes. Such methods are aimed at curtailing the use of hazardous chemicals, reducing waste, and optimizing process conditions. Figure 7 illustrates the diverse strategies employed in sustainable synthesis methods. Consequently, an overview of the most commonly used green and sustainable synthesis methods, including precipitation, sol–gel, solvothermal, and hydrothermal techniques, are provided here.

### 3.1. Sustainable and Green Sol–Gel Synthesis of TiO_2_ Nanostructures

**Figure 8:**
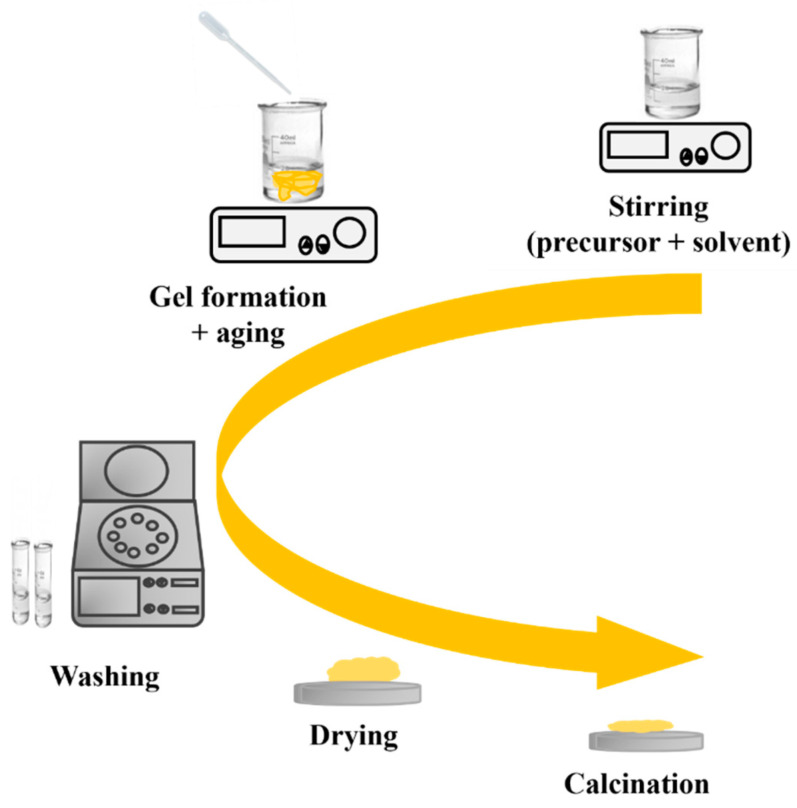
The key processing steps in the sol–gel method.

The sol–gel process is a widely used method for preparing nanostructured MOXs materials. The first work reported on the sol–gel process was by Geffcken and Berger in 1939, and it has become one of the widely utilized methods to produce different MOX nanostructures, such as TiO_2_ [18]. Figure 8 depicts the primary steps of the sol–gel process, including the preparation of a precursor solution, gel formation, and aging. The process starts with the hydrolysis of a solution containing the precursor, which leads to the formation of suspended colloidal particles. The next step is the condensation step, which results in the formation of a gel. Subsequently, the gel is washed, dried, and finally calcinated to generate a solid material [19].

**Figure 9:**
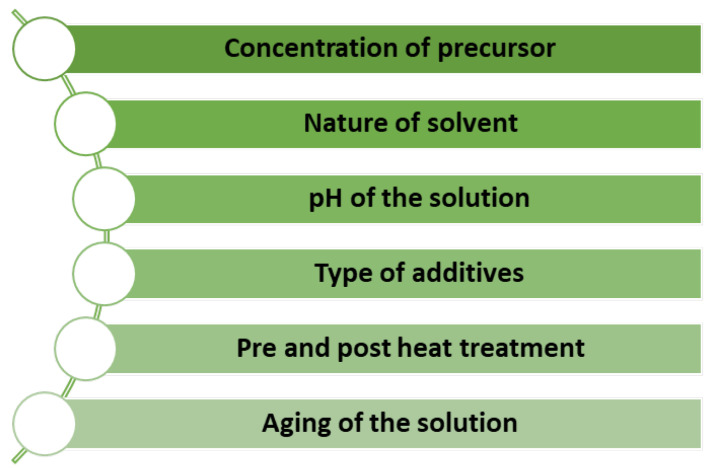
Factors for controlling unique MOX nanostructures by the sol–gel method.

The sol–gel process is cost-effective and has a low waste production, making it an environmentally friendly option. This process also allows the preparation of complex structures and is suitable for large-scale production for a wide range of potential applications [18,20]. Additionally, many parameters are carefully controlled to acquire unique MOX nanostructures, as listed in Figure 9 [21].

In recent years, several research groups have successfully utilized the sol–gel technique to produce different nanostructures of TiO_2_. One of the advantages of using the sol–gel method for preparing TiO_2_ is the ability to prepare TiO_2_ at low temperatures, which reduces the energy consumption and cost of synthesis [22,23,24,25]. However, synthesis of TiO_2_ nanostructures at a low temperature is still progressing with some toxic chemical compounds, such as nitric acid, hydrochloric acid, and ammonia solution. Yang et al. proposed a facile, green, and low-cost approach for the preparation of TiO_2_ via the sol–gel method [26]. The reaction was conducted at a low temperature (30 °C) using less-toxic reagents, such as de-ionized water, acetic acid, and ethanol. The prepared TiO_2_ particles exhibited an amorphous phase and small nanoparticle morphology. Irshad et al. demonstrated the use of non-toxic and less-toxic chemicals, such as 2-propanol, acetic acid, Tween 80, and non-hazardous precursor titanium tetraisopropoxide (TTIP), to prepare TiO_2_ nanoparticles by the sol–gel method [27]. The prepared anatase TiO_2_-NPs (spherical shape) demonstrated a crystallite size of 10–13 nm. In another study, Yazid et al. studied how concentrations of TTIP affected the crystallinity and properties of TiO_2_ thin films deposited through a green sol–gel method at a low temperature (25 °C) [28]. The results showed that higher concentrations of TTIP increased the crystallinity of the mixed phases of TiO_2_ (anatase and rutile), resulting in a crystallite size of approximately 17 nm for anatase and 29 nm for rutile. Furthermore, the surface of the TiO_2_ thin films were found to be cracked with higher concentrations of TTIP.

Consequently, Zou showed the potential of synthesizing TiO_2_ at room temperature (RT) using tributyl titanate as a precursor material, acetylacetone as a stabilizer, and acetic acid and 2-methoxyethanol as co-solvents [29]. This technique combines the advantages of the sol–gel method and the UV illumination process, which results in the hindering of oxygen defects in the TiO_2_ film compared to that synthesised without UV irradiation.

**Figure 10:**
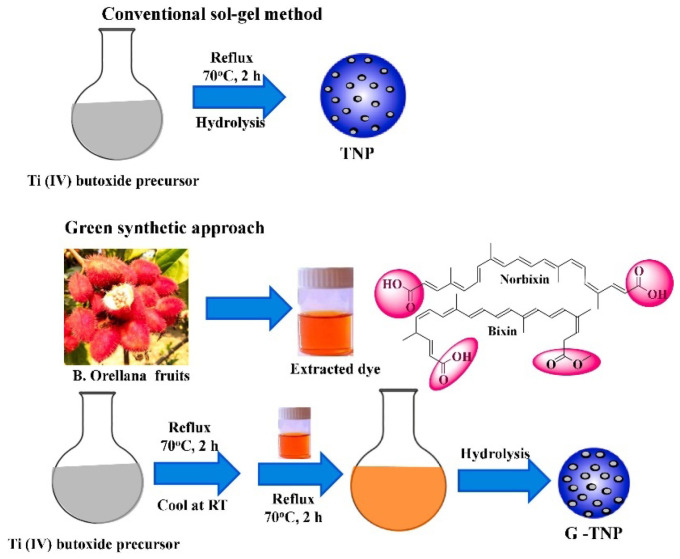
Simplified schematic of the production process of TiO_2_ nanoparticles using the sol–gel method without and with using a bio-capping agent. Reprinted from [30].

On the other hand, employment of a capping agent in the synthesis process reduces agglomeration, which results in a decrement in the average particle size. In this context, the use of bio-capping agents provides an eco-friendly, facile, and one-step synthesis of nanoparticles, which could be easily scaled up to industrial production at a lower cost. For example, Maurya et al. synthesized mesoporous anatase TiO_2_ nanoparticles using the sol–gel synthesis route from titanium (IV) butoxide solution with the help of a bio-capping agent [30]. Furthermore, non-toxic chemicals, such as deionized water, isopropanol, and glacial acetic acid, were used in this work. In Figure 10, the researchers illustrate a comparison of TiO_2_ that is prepared using the same procedures without and with the utilization of extract of bixa orellana seed. Bixa orellana seed extract contains cis-bixin, which can be readily converted to the more stable trans-bixin. The study findings indicated that the TiO_2_ nanoparticles that were prepared, both with and without the bio-capping agent, had a particle dimension of 16 and 13 nm, respectively. However, the crystal phase changed from a mixed phase of brookite and anatase to a pure anatase phase when the green synthesis method was employed.

**Figure 11:**
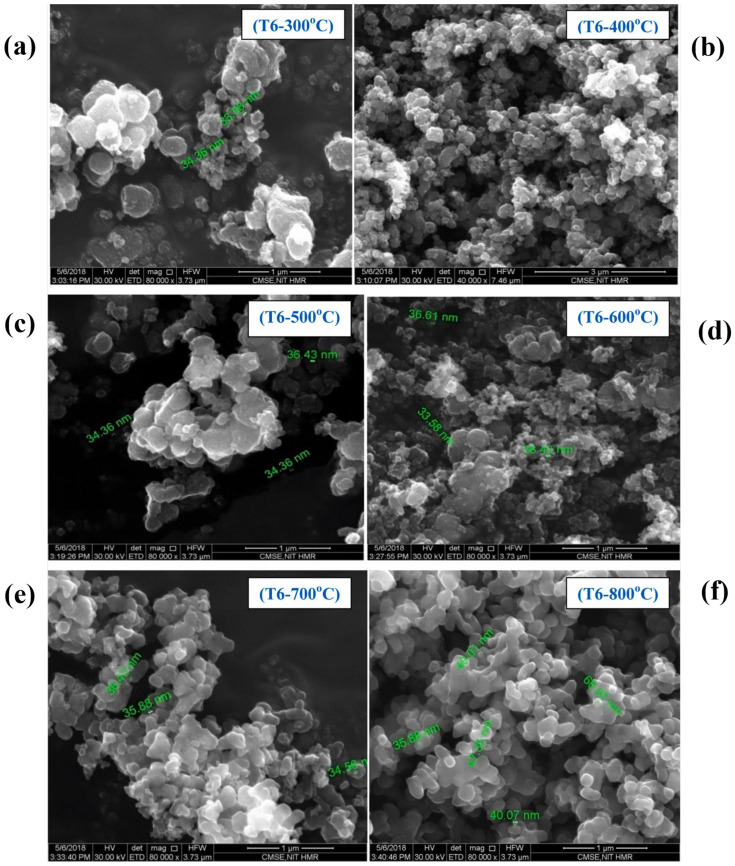
SEM images of TiO_2_ prepared with varying calcination temperatures: (**a**) 300 °C, (**b**) 400 °C, (**c**) 500 °C, (**d**) 600 °C, (**e**) 700 °C, and (**f**) 800 °C at 6 mL of TTIP concentration. Reprinted from [32].

Calcination temperature is one of the crucial parameters that control the morphology and crystallinity of TiO_2_ [31]. For instance, Lal et al. showed the phase transition of TiO_2_ NPs (spherical, Figure 11) from anatase to rutile when the calcination temperature is above the 800 °C [32]. Additionally, their study showed the increment in crystallite size from 7.68 nm to 37.54 nm, along with the phase transition at the optimal TTIP concentration of 6 mL.

**Figure 12:**
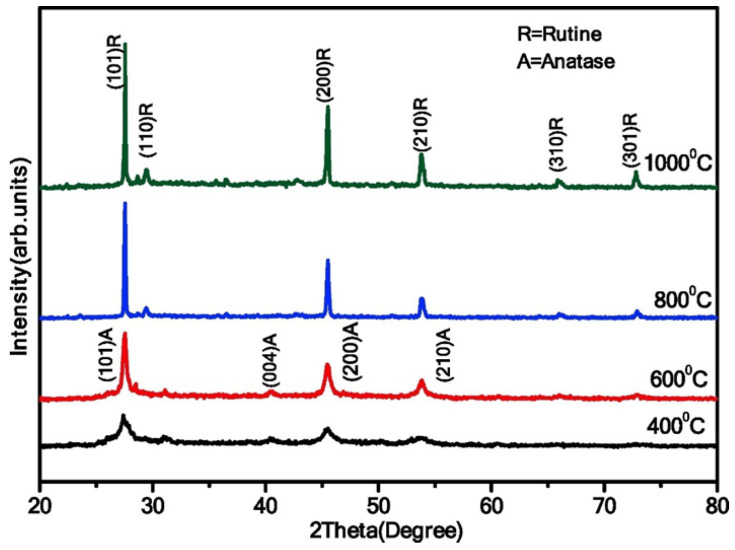
XRD analysis of TiO_2_ NPs synthesized at varying annealing temperatures (400 °C, 600 °C, 800 °C, and 1000 °C). Reprinted from [33].

Similarly, Jule et al. showed a phase transformation from anatase (crystallite size of 15 nm) to rutile (crystallite size of 29 nm) when the calcination temperature increases from 400 to 1000 °C in uniform TiO_2_ NPs (Figure 12) [33]. Additionally, the average size of TiO_2_ NPs increases with increasing annealing temperatures.

In a study conducted by Catauro and colleagues, the effects of heat treatment on the crystal phases and size of TiO_2_ NPs were investigated via the sol–gel method [34]. The sol was divided into two parts and treated differently, with one part (TiO_2_-1) being placed in an oven at 60 °C for 72 h, and the other part (TiO_2_-5) being centrifuged and then treated in a hot muffle furnace at 600 °C for 1 h after three washings with ethanol and water. TiO_2_-1 was further divided into three aliquots and treated at different temperatures, with TiO_2_-2 and TiO_2_-3 being heated at 400 °C and 600 °C, respectively, and TiO_2_-4 being heated at 600 °C after first being treated at 60 °C. The formation of crystal phases was analyzed using XRD measurements, with TiO_2_-1 being found to be amorphous. Partial crystallization occurred in the samples from TiO_2_-2 to TiO_2_-5, with anatase being the only crystal phase detected in TiO_2_-2, and a mixture of anatase and rutile phases being found in the other samples. The SEM images of the TiO_2_ samples treated at different temperatures showed a non-uniform particle size with a high degree of agglomeration. The NPs in the amorphous TiO_2_-1 sample had a size of about 700 nm, while the TiO_2_-4 sample had a smaller particle size (~100 nm) but a higher degree of agglomeration. Accordingly, thermal treatment has a great impact on the crystal growth and morphology of TiO_2_. Furthermore, higher temperatures lead to an increase in particle size and noticeable aggregation of TiO_2_ nanoparticles.

**Figure 13:**
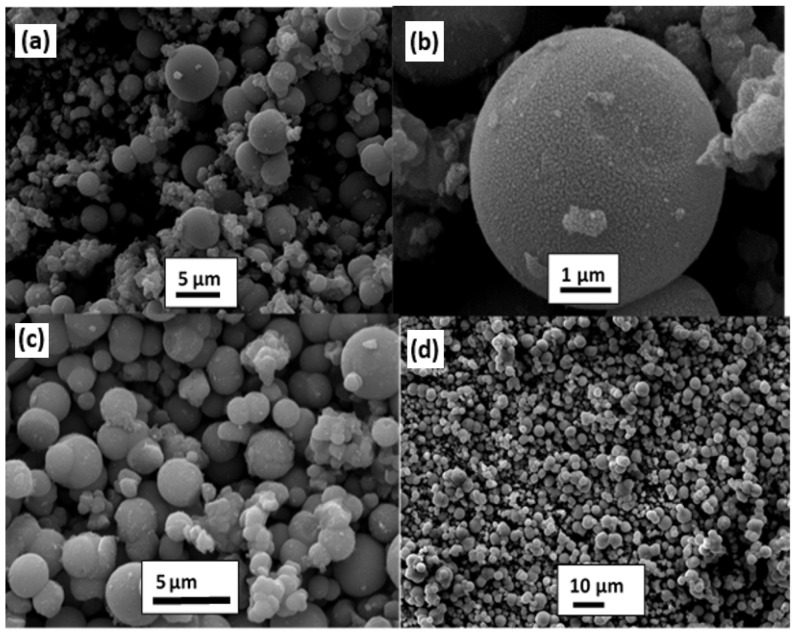
SEM images of (**a**,**b**) TiO_2_, (**c**) Fe-TiO_2_ (0.025 wt%), and (**d**) Fe/TiO_2_ (1 wt%). Reprinted from [35].

Doping is an innovative approach that effectively enhances the characteristics of materials. For instance, Ochoa Rodríguez et al. reported the synthesis of Fe-doped mesoporous TiO_2_ nanoparticles using a simple sol–gel method at low temperatures [35]. Their study focused on investigating the impact of varying iron contents on the synthesized nanoparticles. As shown in Figure 13, the addition of different levels of iron doping does not affect the agglomeration of nanoparticles, leading to similar meso-structures with a spherical morphology and rough surfaces, as well as no cracks. The average sphere size remains within the range of 3–6 μm.

In another study, Zedek et al. showed the potential of using green solvents (ethanol and ethylene glycol) to prepare Fe-doped TiO_2_ NPs via a simple sol–gel method [36]. It was discovered that TiO_2_ NPs, which had been doped with Fe, displayed a tetragonal structure that was a blend of anatase and rutile phases. Additionally, it was noted that Fe (III) was easily integrated into the TiO_2_ lattice without causing any effect on its tetragonal system. In another study, Nithyaa et al. synthesized TiO_2_ and Gd-doped TiO_2_ samples using greener and fewer chemicals by the sol–gel method [37]. The synthesized nanoparticles showed excellent crystallinity, and the diffraction pattern could be compared with the standard anatase phase of TiO_2_. There were no peaks indicating any other crystalline material, and the diffraction pattern of the Gd-doped sample showed a shift indicating the presence of Gd within the Ti-O lattice. The authors also found that pure TiO_2_ and Gd -TiO_2_ particles had a spherical, poly-dispersed shape with irregular morphology, and the addition of Gd did not affect the particles’ shape.

Moreover, co-doping has become a key area of research and is a novel approach that has been gaining significant attention in the field of materials science because it enhances properties when compared to single doping. Hajizadeh-Oghaz used a novel approach to synthesize lanthanum–niobium (La, Nb) co-doped TiO_2_ nanocrystals using the Pechini sol–gel technique [38]. Generally, this process is based on the formation of metal citrate complexes. In this study, polymerization was achieved through the coordination of citric acid and free citric acid via ethylene glycol, resulting in the development of a polymeric resin. This resin served as a steric barrier to facilitate the formation of nanocrystals. The results of the XRD patterns reveal that both undoped TiO_2_ and all (La, Nb)-co-doped TiO_2_-based samples have the anatase-phase crystal structure. The crystal structure type was not affected by the presence of Nb and La in the matrix. Moreover, the size of the anatase-phase crystallite decreases in the (La, Nb)-co-doped TiO_2_ samples compared to undoped TiO_2_. A slight reduction in size was observed with an increase in (La, Nb) content from 1 to 3 mol%. The TiO_2_ particles are uniform in size, ranging from 30 to 40 nm. The TEM image of 2% (La, Nb)-co-doped TiO_2_ nanoparticles displays a similar semi-spherical shape with no highly aggregated particles, with an average particle size of 15–20 nm. Table 1 summarizes the experimental, morphological, and structural characteristics of TiO_2_ nanostructures by the sol–gel method.

### 3.2. Sustainable Precipitation and Green Synthesis of TiO_2_ Nanostructures

**Figure 14:**
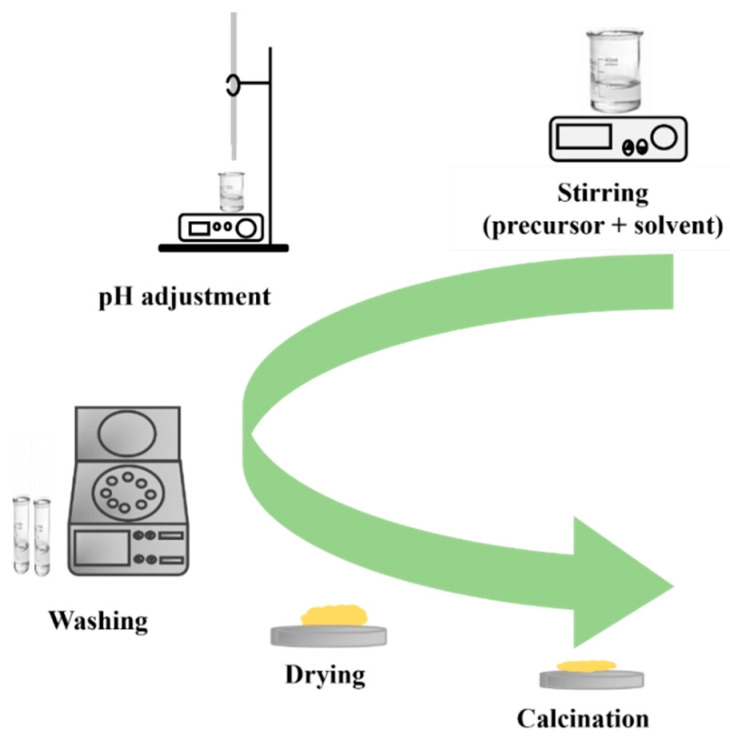
Diagram illustrating the steps of the precipitation method.

The precipitation method is a widely used method for preparing nanostructured materials. The process involves the formation of nanoparticles from a solution through a chemical reaction, resulting in the precipitation of a solid material. In this method, precursors are dissolved in a solvent, and then a reactive agent is added to induce precipitation (Figure 14). The product is often washed, dried, and calcinated to obtain pure nanostructured materials. Calcination removes impurities and stabilizes nanostructures made through the precipitation method, allowing for control over size, shape, and composition.

Concerning sustainable co-precipitation synthesis, which involves the production of nanoparticles without the use of any plant extract, Buraso et al. conducted a study to synthesize TiO_2_ using a low-temperature precipitation method and minimal chemicals, with only titanium isopropoxide as a precursor and deionized water as a solvent [48]. The researchers also investigated the impact of calcination temperature on the crystallinity and morphology of TiO_2_ nanoparticles. The XRD analysis showed that the samples calcined in air at 400 and 500 °C had a pure anatase structure, while those treated at higher temperatures exhibited an anatase–rutile structure. Additionally, as calcination temperature increased from 400 to 700 °C, the size of TiO_2_ nanoparticles increased slightly from 11.3 to 27.4 nm.

Similar, Kalaivani and colleagues utilized the precipitation method to synthesize TiO_2_ nanoparticles and incorporated a green agent, polyvinyl pyrrolidone (PVP), in the synthesis process [49]. This study mainly focused on investigating the impact of calcination temperature on TiO_2_ nanoparticles, ranging from 300 to 600 °C. The XRD analysis of the as-prepared TiO_2_ nanoparticles at various temperatures indicated that both anatase and rutile TiO_2_ nanoparticles possessed a polycrystalline nature with a tetragonal crystal structure. The results also revealed that the average particle size of TiO_2_ increased with temperature. The surface of TiO_2_ nanoparticles was characterized by uniform spherical grains. The sample annealed at 400 °C exhibited a polycrystalline surface with spherical-like structures, having an average particle size of approximately 10 nm. With an increase in the annealing temperature up to 600 °C, the formation of spherical structures was observed, with an average particle size of approximately 22 nm.

Fischer et al. developed a new method for synthesizing TiO_2_ nanoparticles with different crystal phase ratios through low-temperature dissolution–precipitation (LTDRP) on a porous microfiltration membrane made of polyethersulfone [50]. They conducted experiments to determine the effects of acid concentration and temperature on the crystal composition of the synthesized nanoparticles. The amount of hydrochloric acid and the reaction temperature were varied while keeping the concentration of the titanium precursor constant. The researchers found that both acid concentration and temperature had a significant impact on the crystal composition of the synthesized nanoparticles. The shape of TiO_2_ nanoparticles was also affected by the HCl concentration, with spherical and rod-like particles forming at different concentrations. Interestingly, the researchers observed that increasing the HCl concentration resulted in the formation of flower-like structures in rod-like particles. The formation of rutile at higher concentrations of HCl was also confirmed through XRD analysis. Although they presented a new technique, it should be noted that the use of hazardous chemicals, such as HCl, is a potential obstacle to achieving a greener synthesis.

Apart of pure TiO_2_, some researchers investigated the synthesis of doped TiO_2_. For example, Fomekong et al. synthesised undoped and Co-doped TiO_2_ nanoparticles using a co-precipitation method [51]. Cobalt acetate was used as the dopant to obtain 0.5 and 1 mol% of cobalt in TiO_2_. The X-ray analysis showed that the presence of cobalt dopant promoted the conversion of anatase to rutile phase in TiO_2_. The SEM analysis revealed that doping with Co had a noticeable impact on the powder morphology, with the 1% Co-doped TiO_2_ sample displaying larger and well-faceted rhombohedral crystallites with minimal agglomeration. This study provides valuable insights into the effects of Co-doping on the properties of TiO_2_ NPs. Similarly, Ni-doped and undoped TiO_2_ NPs were made using co-precipitation and calcination (3 h at 700 °C) [52]. The prepared pristine TiO_2_ NPs contained both anatase and rutile polymorphs in the synthesized powders, with increasing rutile content as the nickel concentration (0.5, 1.0, and 2.0 mol%) increased. Additionally, spherical NPs (70 nm) of pristine TiO_2_ tended to agglomerate, while Ni doping had a significant impact on the morphology, leading to a lower agglomeration and a smaller particle size with increasing dopant content.

Sangeetha et al. reported low-temperature preparation of bismuth-and-boron-co-doped TiO_2_ nanorod-like structures [53]. Bismuth and boron co-doping was produced using tetrabutyl titanate and boric acid as the source chemicals for Ti, Bi, and B. The final product was crushed and calcined for 4 h at 450 °C. The findings showed that all materials had a highly crystalline anatase structure, with the crystallite sizes ranging from 25 to 34 nm. The morphological analysis showed a spherical shape and rectangular rod-like structures with particle sizes ranging from 50 to 300 nm.

Sharmila et al. reported the synthesis of lithium-activated TiO_2_ nanoparticles using a co-precipitation technique. The precursor materials used were titanium tetrachloride and sodium hydroxide [54]. XRD analysis was carried out to determine the structural characteristics of the synthesized nanoparticles, and the results showed that the crystallite size was in the range of 42 to 52 nm. The majority of the TiO_2_ nanocrystals were found to be in the rutile crystal phase with some traces of a brookite phase. The SEM results revealed that the nanoparticles had a rectangular and hexagonal morphology.

Using sodium hydroxide (NaOH) as a precipitator can significantly enhance the sustainability of the precipitation process [55], especially when compared to other reports with ammonia [56,57]. While some studies still relied on ammonia as a precipitating agent, this compound can be highly volatile and corrosive, making it less environmentally friendly and potentially hazardous to handle. On the other hand, sodium hydroxide is a readily available and inexpensive chemical that can effectively precipitate a wide range of compounds and poses less of a risk to human health or the environment. Therefore, the use of sodium hydroxide as a precipitator can be a promising approach for making chemical synthesis more sustainable and reducing the overall environmental impact of the process.

Utilizing plant extracts as green agents is another interesting approach to secure the sustainability of production. However, these methods still incorporate toxic chemicals in the synthesis process. It is worth pointing out that water or an organic solvent is commonly used to extract phytochemicals, and the process is carried out at temperatures not exceeding 100 °C. For example, Ansari et al. introduced a novel and ingenious method to produce TiO_2_ NPs at low temperatures that is sustainable [58]. Their process employs *Acorus calamus* (*A. calamus*) leaf extract as a new biogenic source, as well as a capping and reducing agent with ammonia as a precipitator.

**Figure 15:**
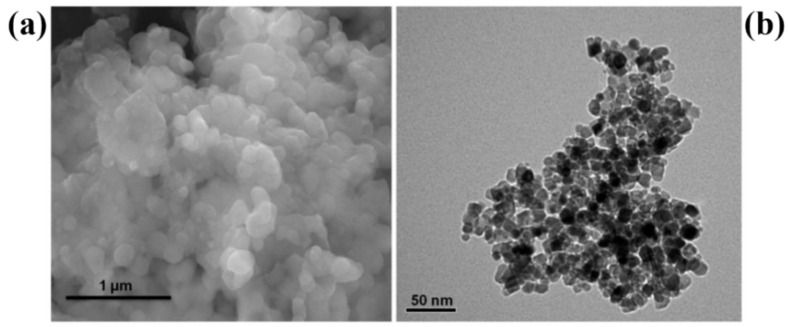
Green synthesis of TF-TiO_2_ NPs: (**a**) HR-SEM and (**b**) HR-TEM. Reprinted from [59].

On the other hand, a study conducted by Subhapriya et al. investigated a method for the chemical and green synthesis of TiO_2_ using extract from the leaves of *Trigonella foenum* (TF) [59]. One noteworthy aspect of this research is the use of sodium hydroxide (NaOH) as a precipitator. Their results, as shown in Figure 15, indicate that nanoparticles produced using a plant extract exhibit even dispersion on the surface, which is accompanied by the development of aggregated nanoparticles with a clear view of spherical nanoparticles. Moreover, the chemical and green synthesis method yielded well-crystalline titanium with an anatase phase.

**Figure 16:**
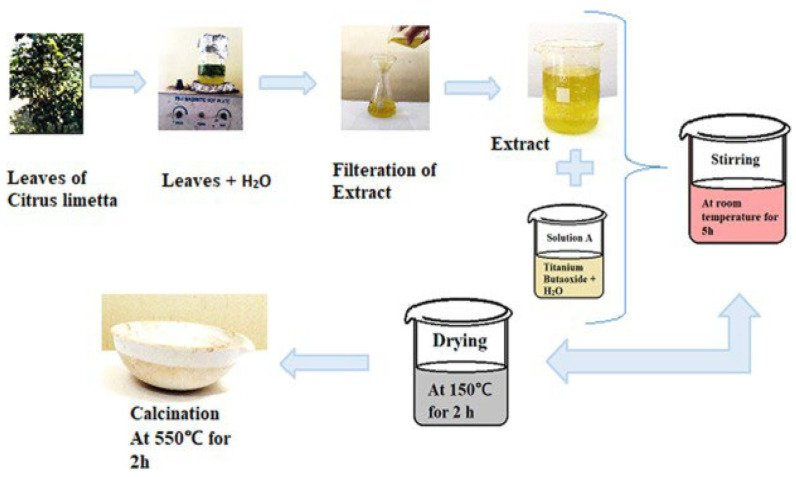
Flowchart of the synthesis of TiO_2_ nanoparticles. Reprinted from [60].

Nabi et al. prepared TiO_2_ NPs using a cost-effective and room-temperature green synthesis method with the extract of *Citrus limetta* (Figure 16) [60]. Titanium butoxide was added to distilled water and mixed with the extract. The synthesized TiO_2_ had an anatase crystalline phase. Furthermore, the nanoparticles were spherical in shape and had a uniform distribution, with a particle size of 80–100 nm.

In another study, reddy et al. prepared flower-like TiO_2_ nanoparticles via a novel green approach using medicinal plant leaf extracts, namely *Ocimum tenuiflorum* plant (OTP) and *Calotropis gigantea* plant (CGP), at room temperature [61]. The plant leaf extracts, which are rich in secondary metabolites, such as polyphenols, flavonoids, alkaloids, terpenoids, and peptides, contain hydroxyl and ketonic groups that help in reducing Ti^4+^ ions to TiO_2_ state, as well as in green stabilizing and capping nanoparticles. The XRD results indicated that the OTP extract produced TiO_2_ NPs in the anatase phase, while the CGP extract produced TiO_2_ NPs in the rutile phase. Furthermore, the TiO_2_ produced from the CGP extract displayed a higher level of crystallinity, with a smaller crystallite size. The SEM and TEM results revealed that the OTP sample had spherical-shaped grains with an average size of 100 nm, while the CGP sample had flower-like shaped grains with an average size of 200 nm. Additionally, reddy et al. synthesized TiO_2_ NPs using an environmentally friendly and eco-sustainable approach [62]. This approach involved the use of TiCl_4_ as a precursor and the utilization of C. gigantea (CG) plant leaf extract as a catalyst. The XRD analysis results indicated that the TiO_2_ NPs in the rutile phase had a tetragonal structure, with a crystallite size calculated to be 9.84 nm. Furthermore, the SEM and TEM images reveal a non-uniform distribution of spherical- and flower-like shaped grains, with an average grain size of 100 nm.

A similar study was reported by Prashanth et al., in which they prepared TiO_2_ using *Calotropis gigantea* (CG) leaf extract and at room temperature [63]. Titanium isopropoxide and the CG leaf extract were added to distilled water. Their results showed spherical, dispersed anatase TiO_2_ with an average particle size of approximately 42 nm. Hariharan et al. reported a fast green method with easy, minimal chemicals at low temperature (50 °C) to prepare TiO_2_ using *Cynodon dactylon* leaves [64]. The researchers indicated that the NPs were hexagonal in shape and had a size range of 13 to 34 nm. Furthermore, the TiO_2_ NPs were found to be in the anatase phase.

**Figure 17:**
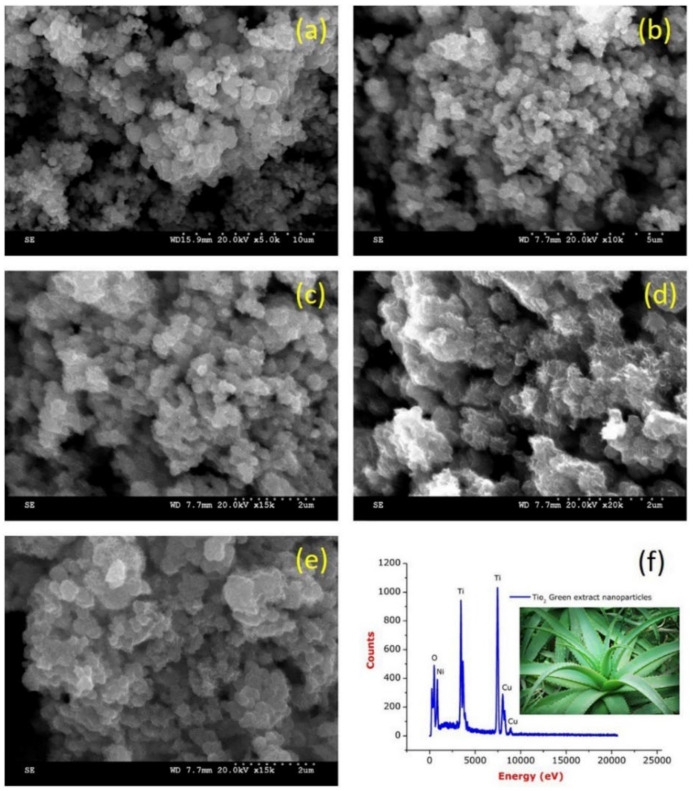
(**a**–**e**) FESEM images of TiO_2_-NPs and (**f**) EDS spectrum of TiO_2_-NPs. Reprinted from [65].

Rajkumari et al. produced TiO_2_ NPs by green synthesis using minimal chemicals and *Aloe barbadensis* as a reducing and stabilizing agent [65]. The results of the study, as demonstrated in Figure 17a–e, show that the TiO_2_ NPs are poly-dispersed and have a spherical shape, with a size range of 20 to 50 nm. Additionally, the EDS analysis results, depicted in Figure 17f, reveal sharp and intense peaks of titanium (Ti) and oxygen (O), indicating a high concentration of titanium and oxide peaks. This confirms that the synthesized TiO_2_ NPs are of high purity.

**Figure 18:**
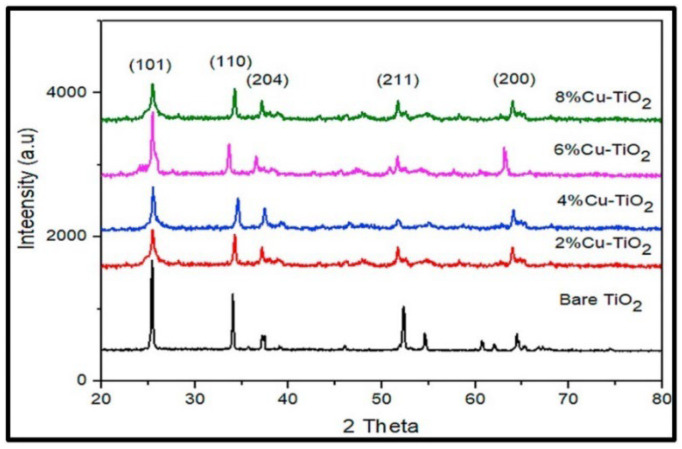
Comparative X-ray diffraction analysis of pure TiO_2_ and Cu-Doped TiO_2_ NPs at varying concentrations. Reprinted from [66].

Aside from pure TiO_2_, various research groups have utilized a green approach to synthesize doped TiO_2_ nanostructures. Ramzan et al. reported the synthesis of Cu-TiO_2_ nanoparticles (NPs) using *Cedrus deodara* extract by a low-temperature method (65 °C) [66]. They found that the particles agglomerated. The average size of the Cu@TiO_2_ nanocomposites was determined to be around 10.01 ± 0.30 nm. Figure 18 shows the XRD results of Cu@TiO_2_ NPs, indicating the presence of distinct diffraction peaks located at 27.5°, 36.2°, 41.3°, 44.5°, 54.30°, 57.6°, and 64.8°, which have been indexed to planes 101, 110, 204, 120, 211, 220, and 200, respectively. The highest peaks are attached to tetragonal structure and anatase phase.

**Figure 19:**
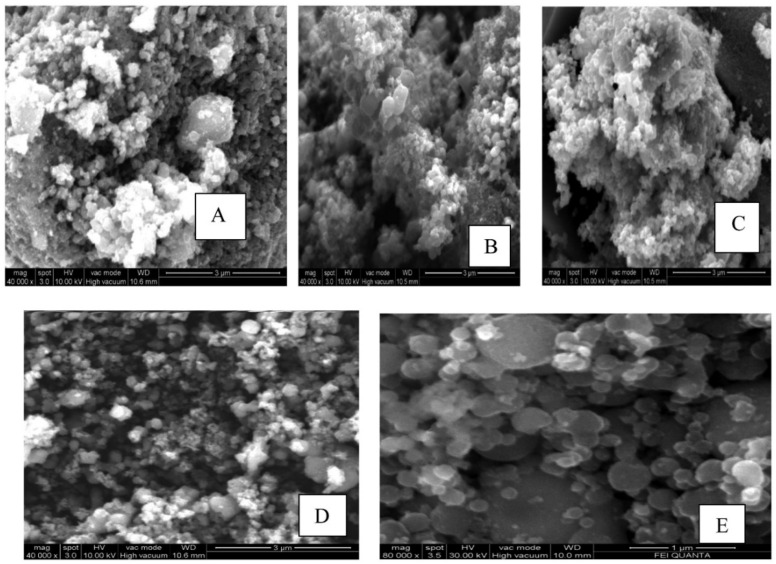
SEM images of (**A**) PB-TiO_2_, (**B**) OT-TiO_2_, (**C**) MO-TiO_2_, (**D**) CS-TiO_2_, and (**E**) TiO_2_. Reprinted from [67].

Pushpamalini et al. conducted a study on the green synthesis of TiO_2_ NPs using four different leaf extracts as reducing agents [64]. The leaf extracts of piper betel (PB), *Ocimum tenuiflorum* (OT), *Moringa oleifera* (MO), and *Coriandrum sativum* (CS) were used to synthesize TiO_2_ from titanium tetraisopropoxide. As shown in Figure 19, the TiO_2_ NPs are nearly spherical and formed in clusters. The SEM image of MO-TiO_2_ shows many aggregated particles, confirming the greater influence of moringa leaf extract on the structure of nano-TiO_2_. The XRD pattern reveals that only anatase-phase TiO_2_ resulted during the green synthesis, while the chemical method resulted in both anatase and rutile phases. The particle size of five different types of TiO_2_, namely TiO_2_, PB-TiO_2_, OT-TiO_2_, MO-TiO_2_, and CS-TiO_2_, was found to be 24, 6.4, 7.0, 6.6, and 6.8 nm, respectively. Table 2 summarizes the reported TiO_2_ nanostructure using green methods.

### 3.3. Sustainable and Green Hydro/Solvo-Thermal Synthesis of TiO_2_ Nanostructures

#### 3.3.1. Hydrothermal Synthesis

**Figure 20:**
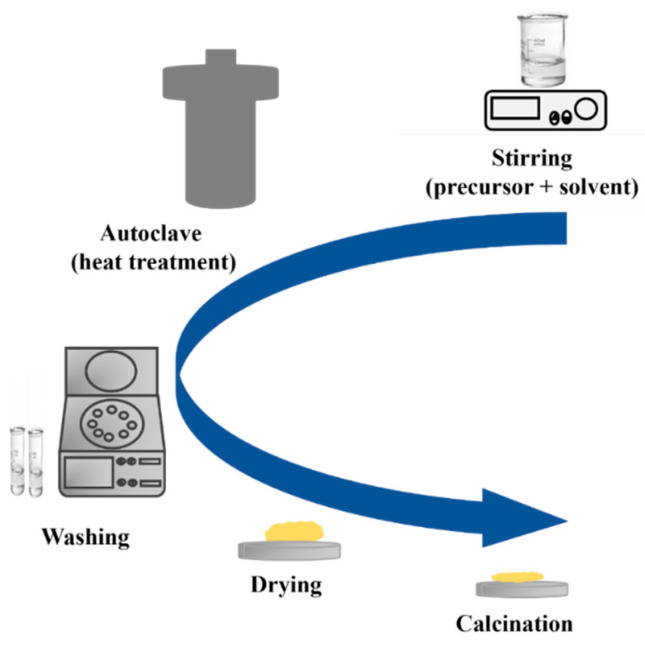
Schematic diagram of a hydrothermal method.

In recent years, hydrothermal synthesis is a method widely used to prepare various nanostructures of TiO_2_, such as quantum dots [72], nanowires (NWs) [73], nanotubes (NTs) [74], nanosheets (NSs) [75], and others. The hydrothermal method is divided into two categories based on the solvent solution used: acid-hydrothermal and alkali-hydrothermal methods. As shown in Figure 20, the process begins by mixing the precursor and solvent to form a homogeneous mixture. The mixture is then subjected to thermal treatment using a closed system in a Teflon autoclave under a specific pressure and temperature. The use of a closed system with controlled temperature and pressure offers a higher level of stability in the process. This stability leads to better control over the size of crystals, the initiation of nucleation, and the degree of crystallinity achieved [76]. The resulting product is then washed and dried, followed by crystallization through annealing at a specific temperature [77].

Additionally, researchers have developed a hydrothermal procedure that uses ultrasonic energy and autoclave (ultrasonic-assisted hydrothermal) to produce more precise and controlled nanoparticles with regulated size and shape [78]. The ultrasonic treatment leads to reduced particle deagglomeration and smaller particle sizes, resulting in higher phase purity. Microwave-assisted hydrothermal synthesis is also a modern method that utilizes the properties of microwaves to enhance the hydrothermal process. This results in rapid and uniform heating, leading to high-quality products being formed in a shorter period compared to traditional hydrothermal synthesis. The advantage of this method lies in its faster reaction rates and uniform heating, thereby enhancing overall efficiency. Low-temperature hydrothermal synthesis offers numerous benefits, including sustainability and cost-effectiveness [79,80,81].

Green hydrothermal synthesis using plants extracts represents a promising, sustainable, and eco-friendly alternative to traditional material synthesis methods [82]. Several research efforts have been directed toward exploring advanced hydrothermal techniques, including ultrasonic hydrothermal, microwave hydrothermal, and low-temperature hydrothermal methods, to ensure a green, eco-friendly, and sustainable approach in the preparation of TiO_2_ nanostructures (Table 3). 

**Figure 21:**
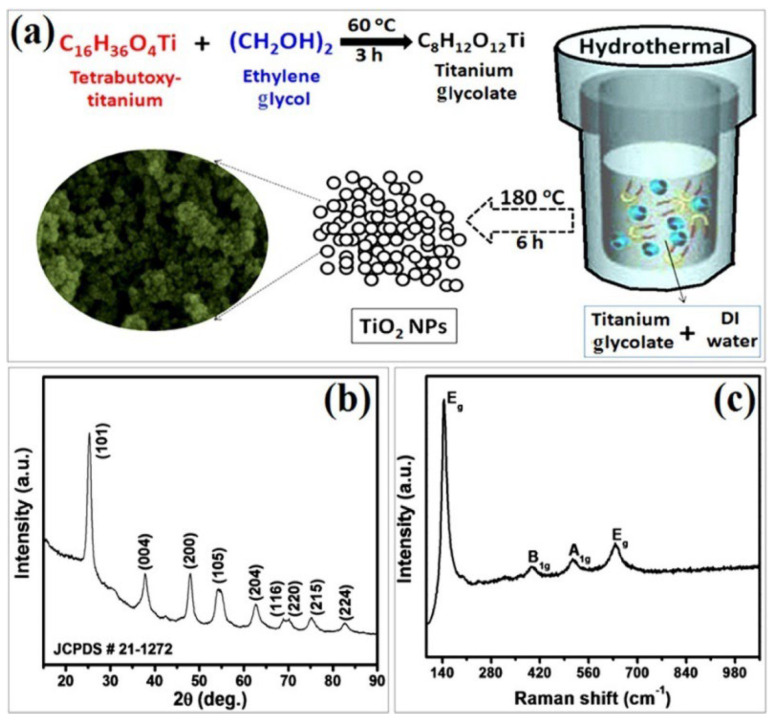
Synthesis and characterization of synthesized anatase TiO_2_ NPs: (**a**) synthesis scheme, (**b**) XRD, and (**c**) Raman spectrum of TiO_2_. Reprinted from [81].

In this context, a simple hydrothermal approach was applied for a large-scale synthesis of anatase TiO_2_ NPs by Navale et al. using titanium glycolate as the precursor [81]. As shown in Figure 21a, this precursor is produced from mixing ethylene glycol and tetra-butoxy titanium. Their XRD findings indicate the presence of pure, tetragonal anatase phase of TiO_2_ nanoparticles (Figure 21b), with a crystallite size of approximately 14 nm. The Raman analysis displayed in Figure 21c exhibits four active Raman peaks at 144, 397, 514, and 638 cm^−1^. The peak observed at 144 cm^−1^ is highly intense, while the peaks situated at 397 and 514 cm^−1^ correspond to the B_1g_ and A_1g_ vibration modes, respectively. The peaks at 144 and 397 cm^−1^ signify the involvement of the O-Ti-O bending vibration. Meanwhile, the peaks at 514 and 638 cm^−1^ are related to the Ti-O stretching vibration.

**Figure 22:**
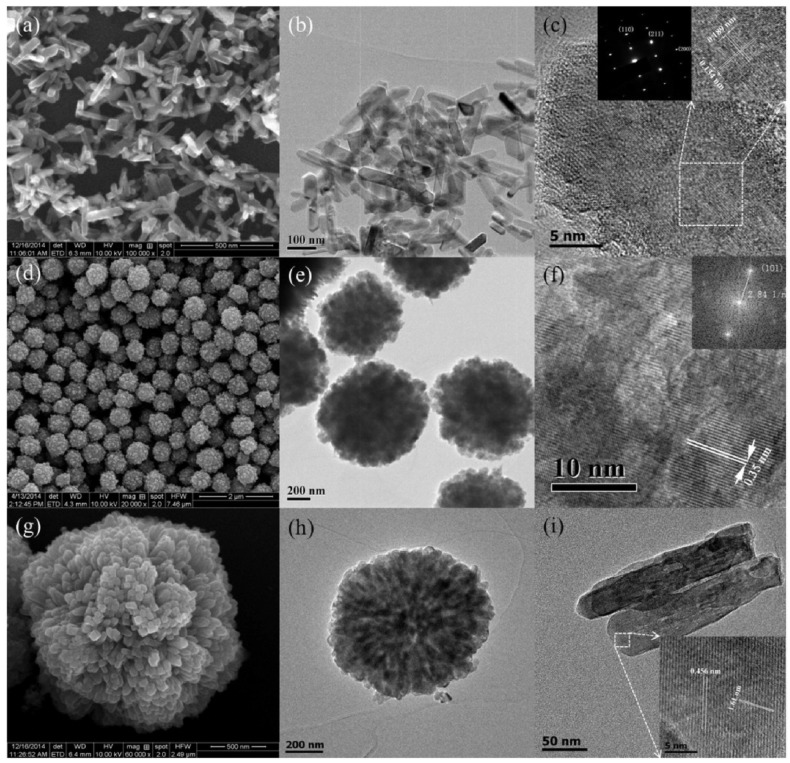
SEM and TEM characterizations of TTIP/OA samples with varied ratios: (**a**–**c**) 2:1, (**d**–**f**) 1:3, and (**g**–**i**) 1:6. Reprinted from [83].

Wang et al. showed the tunability of the morphologies and phases of TiO_2_ NPs using a green hydrothermal approach [83]. Oxalic acid (OA) was dissolved in H_2_O to form a transparent solution. OA, a biocompatible reagent commonly found in organisms and plants, is known for its ability to chelate with metal ions. As such, it was anticipated that OA could regulate the hydrolysis of TTIP, allowing control over the morphology and microstructure of the resulting crystal products. Therefore, their study focused on examining the impact of varying the molar ratio of TTIP/OA, ranging from 2:1 to 1:1, 1:3, 1:6, and 1:9. Figure 22 illustrates how the molar ratio of TTIP/OA affects the morphology and crystal phase of TiO_2_ nanoparticles. With the addition of a small amount of oxalic acid, rod-shaped nanoparticles with a thickness of 15 nm and a length of 150 nm are obtained, exposing mainly the (110) facet (Figure 22a–c). Increasing the TTIP/OA ratio results in uniform sphere-shaped aggregates of nanoparticles around 20 nm in size, forming mesocrystals (Figure 22d–f). At higher OA concentrations, dandelion-like assemblies composed of nanorods around 200 nm in length and 50 nm in thickness are synthesized, with lattice distances corresponding to the rutile phase (Figure 22g–i).

Nagaraj et al. synthesized silver-coated TiO_2_ nanostructures (Ag@TiO_2_) using extract of leucas aspera plant [84]. The *Leucas aspera* leaf extract was used as a reducing and capping agent through the biological reduction of silver nitrate. Their research centred around the use of different amounts of silver. As a results, they found that the surface of TiO_2_ NPs underwent a transformation after the deposition of various Ag amounts. As the Ag amounts were modified, the morphologies of the resulting Ag@TiO_2_ nanostructures also altered. Besides, hydrothermal treatment led to the formation of rod-shaped TiO_2_ NPs and small, spherical Ag NPs. The researchers determined that the doping concentration and the hydrothermal method of preparation were the two primary factors impacting the morphological changes.

In a study by Hariharan et al., TiO_2_ NPs were synthesized using aloe vera gel (AV) as a capping agent via a hydrothermal approach [85]. A preparation of aloe vera gel extract was conducted using deionized water as a solvent. In this solvent, titanium (IV) isopropoxide was introduced and subsequently subjected to the hydrothermal treatment. The researchers found that the AV-TiO_2_ structure is composed of an anatase phase with a crystallite size of 9 nm. In terms of morphology, the material displays a tetragonal shape. The particle size ranges from 6 nm to 13 nm, with only slight variations in size.

**Figure 23:**
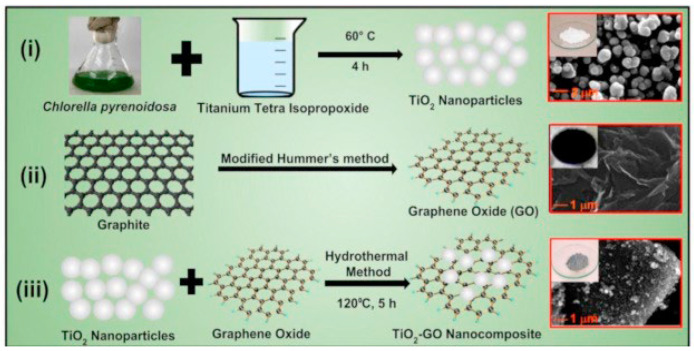
Synthesis process for (**i**) TiO_2_ NPs using green alga *Chlorella pyrenoidosa*, (**ii**) GO, and (**iii**) TiO_2_-GO nanocomposite using a hydrothermal method. Reprinted from [86].

In another work, Sharma et al. reported a novel method for synthesizing TiO_2_ NPs using green alga *Chlorella pyrenoidosa* (as a reducing and capping agent) [86]. The synthesized TiO_2_ nanoparticles were then deposited onto graphene oxide (GO) sheets to form a TiO_2_-GO nanocomposite. Figure 23 presents the synthesis steps and the morphology of (a) TiO_2_ nanoparticles, (b) graphene oxide, and (c) TiO_2_-GO nanocomposite. From the SEM analysis, it was determined that the spherical TiO_2_ nanoparticles had a diameter of approximately 50 nm. The GO sheets, on the other hand, appeared wrinkled and were several micrometers in size. Upon studying the TiO_2_-GO nanocomposite, it was found that the TiO_2_ NPs tended to accumulate along the wrinkles and edges of the GO sheets. Despite this, the spherical morphology of the TiO_2_ NPs remained largely unaffected after being coupled with GO. The XRD analysis confirmed a pure tetragonal anatase phase, while the TiO_2_-GO nanocomposite showed mostly anatase TiO_2_ and GO (with a lower intensity), indicating a disruption of the regular GO stack by the intercalation of TiO_2_ NPs. Table 3 summarizes the experimental, morphological, and structural performances of reported TiO_2_ nanostructures using the hydrothermal method.

**Table 3:** A summary of synthesized TiO_2_ nanostructures using the hydrothermal method.

Chemicals	Synthesis Conditions	Structural and Morphological Properties	Ref.
Titanium glycolate Distilled water	Autoclave temp: 180 °C—Time: 6 h Washing: distilled water Drying- Temp: 60 °C—Time: 2 h	Phase: tetragonal anatase Morphology: aggregates, composed of NPs	[81]
Titanium butoxide Silver nitrate Trini-nitrophenol nitric Hydrochloric acids DD water Leucas aspera plant	Plant Stirring: plant + DD water Time: 30 min—Stored at temp: 5 °C TiO_2_ preparation Stirring time: 2 h—Temp: RT Autoclave temp: 160 °C—Time: 15 h Drying temp: 110 °C—Time: 10 h	Morphology: spherical shape Particle size: 5 nm	[84]
Deionized water Aloe Vera gel TTIP	Plant ectract Stirring: aloe vera gel + DI water Time: 1 h—Temp: 20 °C TiO_2_ preparation Autoclave temp: 180 °C—Time: 4 h Drying temp: 80 °C Calcination temp: 500 °C—Time: 5 h	Phase: anatase Morphology: nanoparticles Particle size: Around 6 to 13	[85]
Algal powder Graphite powder TTIP Ethanol DI water	Plant extract Stirring: algal powder + DI water Temp: 70 °C—Time: 30 min—Then filtered TiO_2_ nanoparticle preparation Stirring: algal extract + TTIP Temp: 60 °C—Time: 4 h Washing: DI water Drying temp: 60 °C—Time: 12 h Annealing temp: 600 °C—Time: 2 h TiO_2-_GO nanocomposite preparation Ultrasonic treatment: graphene oxide + DI water + ethanol Time: 1 h Stirring: TiO_2_ NPs + GO—Time: 2 h Autoclave temp: 120 °C—Time: 5 h Washing: DI water + ethanol Drying temp: 60 °C—Time: 15 h	TiO_2_ Phase: tetragonal anatase Morphology: spherical nanoparticles Diameter: 50 nm TiO_2-_GO Morphology: TiO_2_ nanoparticles + GO sheets	[86]
Tetrabutyl orthotitanate Acetylacetone Millipore water Ammonia solution	Autoclavev temp: 170 °C—Time: 24 h Washing: HCl, 2-propanol, and Millipore water Drying temp: 120 °C—Time: 12 h Calcination temp: 450 °C—Time: 1 h	Phase: anatase Morphology: nanorods (NRs) Length of NRs: 100 nm Diameter of NRs: 30 nm	[87]
Titanium tetraisopropoxide Deionized water NaOH	Stirring time: 3 h Autoclave temp: 180 °C—Time: 24 h Washing: distilled water and ethanol Drying temp: 80 °C—Time: 3 h Annealing temp: 400 °C—Time: 5 h	Phase: anatase Crystallite size: 14 nm (pH = 7) and 16 nm (pH = 9) Morphology: nanorods with rod-like morphology (pH = 7) nanoplatelet-like structure (pH = 9)	[88]
TTIP HNO_3_ NaOH	Stirring temp: 80 °C—Time: 6 h Autoclave temp: 150 °C—Time: 60 min Washing: water (Acidic medium) Washing: HCl (alkaline medium) Drying temp: 60 °C Note: Teflon autoclave placed inside a modified domestic microwave oven	Acidic medium Phase: anatase + brookite + rutile Crystallite size: 6.0 nm Morphology: NPs Particle size: 10 and 15 nm Alkaline medium Phase: anatase + brookite Morphology: irregular spheres	[89]
TTIP Ethanol Distilled water	Stirring time: 30 min Ultrasonic bath time: 20 min Autoclave temp 150 °C—Time: 3 h Washing: deionized water Drying temp: 110 °C—Time: 5 h Calcination temp: 500 °C—Time: 2 h	Phase: tetragonal crystal structure Crystallite size: range of 31–42 nm Morphology: spherical nanoparticles Particle size: range of 32–48 nm	[90]
C_12_ H_28_ O_4_ Ti C2 H_7_ NO_3_ NaOH	Autoclave temp: 150 °C—Time: 20 h Washing: distilled water Drying temp: 60 °C—Time: 24 h—Treated with HCl for 1 h Washing: distilled water and ethanol Drying temp: 100 °C—Time: 1 h Calcination temp: 500 °C—Time: 1 h	Phase: anatase + rutile Morphology: nanorod-like structure Diameters: 30–50 nm Lengths: ≈1825 nm	[91]
Titanium powder Distilled water	Autoclave temp: 75 °C—Time: 4 h Washing: ethanol and water Calcination temp: 500 °C—Time: 3 h	Phase: tetragonal Morphology: semi-spherical NPs Particle size: ≥50 nm	[92]
TTIP Cobalt nitrate Glacial acetic acid Ethanol Distilled water	Autoclave temp: 120 °C—Time: 5 h Washing: ethanol Drying temp: 60 °C Calcination temp: 350 °C—Time: 2 h	Morphology: nanoparticles	[93]
*M. citrifolia* extract Ethanol TiCl_4_	Plant extract Stirring: leaves of *M. citrifolia* + ethanol Time: 20 min—Temp: 50 °C—Stored at temp of 4 °C TiO_2_ preparation Autoclave temp: 120 °C—Time: 8 h Drying temp: 100 °C—Time: 4 h	Phase: tetragonal crystal structure Crystallite size: ≈10 nm Morphology: quasi-spherical NPs Particle size: from 10 to 20 nm	[94]
TTIP Distilled water Aloe vera gel Silver nitrate	Plant extract Stirring: aloe vera gel + DI water Time: 2 h—Temp: 90 °C—Stored at temp of 4 °C TiO_2_ preparation Stirring time: 1 h Autoclave temp: 180 °C—Time: 24 h Drying temp: 120 °C—Time: 2 h Calcination temp: 500 °C—Time: 5 h	Morphology: nanorods and nanoparticles	[95]

#### 3.3.2. Solvothermal Synthesis

Solvothermal and hydrothermal syntheses are similar processes, but solvothermal methods utilize aqueous solutions of organic compounds as the solvents, such as ethanol and ethylene glycol, while hydrothermal synthesis exclusively uses water as the solvent [17]. The use of an organic solvent allows for greater control over the reaction conditions, making solvothermal synthesis a versatile and widely used technique for synthesizing nanomaterials. Additionally, an organic solvent can also play a role in the formation of the final product, making it an important factor in the outcome of the synthesis process. Furthermore, the use of non-toxic solvents and non-corrosive solutions in green solvothermal synthesis minimizes harm to the environment and conserves raw materials, making it a sustainable and eco-friendly method.

**Figure 24:**
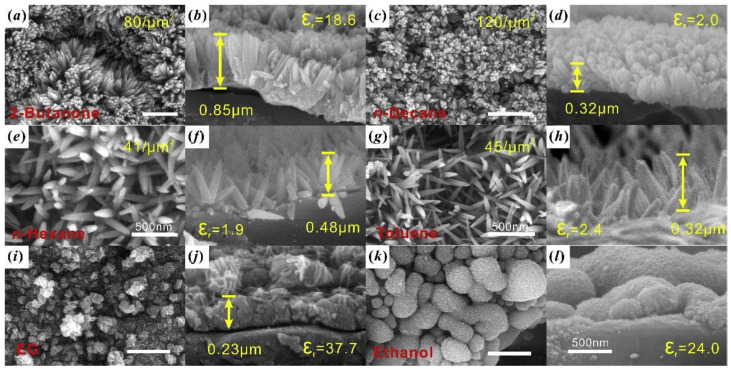
Comparative SEM analysis of TiO_2_ nanostructures using various organic solvents: (**a**,**b**) 2-butanone, (**c**,**d**) *n*-decane, (**e**,**f**) *n*-hexane, (**g**,**h**) toluene, (**i**,**j**) ethylene glycol, and (**k**,**l**) ethanol. Reprinted from [96].

Lu et al. conducted an experiment to explore how different solvents affect the solvothermal synthesis of TiO_2_ nanoarrays on cordierite monolithic substrates [96]. They chose six organic solvents and discovered that the dielectric constant of the solvents plays a vital role in determining the TiO_2_ nanoarrays’ morphology and phase. The solvents with moderate dielectric constants can dissolve partially in the aqueous phase, impacting the reaction rate and promoting heterogeneous growth. The resulting samples show that 2-butanone, n-decane, n-hexane, and toluene produce nanoarray bundle structures with vertically aligned nanorods and a rutile phase. In contrast, ethylene glycol and ethanol produce nanosheet layers and nanoparticle films, respectively (Figure 24), with an anatase phase. This study emphasizes the importance of solvent selection in controlling a sample’s structure and phase. The best reactant combination is 2-butanone and TBOT, resulting in uniform nanoarrays. The researchers also noted that 2-butanone and ethylene glycol are considered green solvents due to their relatively low toxicity compared to other solvents.

In another study, Alosfur et al. synthesized TiO_2_ nanorods by utilizing TTIP as the precursor and ethylene glycol as the solvent in a solvothermal process at low temperatures of 100, 140, and 180 °C [97]. The resulting sample was found to be composed of pure anatase-phase TiO_2_ in a nanorod shape. However, an increase in synthesis temperature above 140 °C led to a decrease in specific surface area.

Huang et al. synthesized TiO_2_ using a simple solvothermal method with different solvents, including ethanol (EA), ethylene glycol (EG), and glycerol (GL). The XRD analysis of the TiO_2_ samples prepared with different solvents revealed that all samples, before and after calcination, exhibited the characteristic peaks of anatase TiO_2_. Interestingly, TiO_2_-EA before calcination and EA-TiO_2_ after calcination also showed weak peaks corresponding to the brookite crystal plane. In terms of morphology, the TiO_2_ remained irregular and lump-like regardless of the solvent used. This suggests that the solvent employed does not have a significant impact on the shape of TiO_2_ in this context. However, particle size varies depending on the solvent used [98].

Use of hazardous hydrofluoric acid (HF) in the preparation of surface-fluorinated anatase TiO_2_ nanosheets is a major obstacle for their commercialization. To address this issue, a new facile, green, and fluorine-free solvothermal synthesis method has been developed by Zulfiqar et al. [99]. In this method, N, N dimethylformamide (DMF) was used as the morphology-controlling agent under an alkaline environment. This approach provides an environmentally friendly alternative to the traditional synthesis methods using HF.

## 4. Chemical/Gas-Sensing Applications

Gas sensing plays a crucial role in the monitoring of air quality and detection of harmful gases in the environment. In recent years, TiO_2_ nanostructures have garnered considerable interest in the gas-sensing arena due to their distinctive physical and chemical properties. The numerous publications on TiO_2_ in various research fields over the past five years attest to their potential. Despite this potential, the widespread utilization of 2D TiO_2_ nanostructures in gas sensing is still limited. This section aims to give a comprehensive overview of recent advancements in TiO_2_ nanostructure applications for gas sensing, emphasizing the benefits and limitations of this promising technology.

### 4.1. Pristine Nanostructures

Zero-dimensional TiO_2_ nanostructures, such as quantum dots, nanospheres, and nanoparticles, are regarded as the top choice for miniaturized and ultrasensitive chemical reactions due to their ample specific surface area, which provides abundant adsorption sites for gases [100]. For instance, Sugahara and colleagues demonstrated impressive gas-sensing response and recovery times (1 s/1 s) toward propylene glycol at 350 °C with anatase TiO_2_ nanospheres, which was attributed to their extensive surface area and highly crystalline structure [101]. Additionally, TiO_2_ nanoparticles were shown to have a low detection limit of 500 ppb when detecting acetone at 250 °C, thanks to their high surface area of 25.2169 m^2^.g^−1^ and an average pore diameter of approximately 25 nm. Table 4 summarizes the gas-sensing performance of 0D TiO_2_ in recent publications.

The utilization of 1D TiO_2_ structures, such as nanowires and nanotubes, in gas sensing has demonstrated promising outcomes and can be optimized for even better performance (Table 5). To this end, innovative techniques, such as doping, decoration, and heterostructures, have been introduced to augment the capabilities of 1D TiO_2_ gas sensors. These sensors have a wide range of potential applications, ranging from environmental monitoring to industrial process control and health and safety. This highlights the significance of 1D TiO_2_ gas sensors as a promising technology for sensing various gases, including volatile organic compounds (VOCs), nitrogen oxides (NOx), and others [112,113,114,115,116].

Typically, the response of sensors intensifies as the concentration of the target gas increases. The surface state, morphology, catalytic activity, and spillover effect also play crucial roles in enhancing the key functions of a sensor, including its response. For instance, TiO_2_ nanotree arrays exhibit exceptional ethanol-sensing response (9.2) compared to TiO_2_ nanowires due to their larger specific surface area and resultant active sites, accessible channels for gas flow, and potential barriers established by phase junctions at the trunk/branch interface [117]. Similarly, Tshabalala et al. demonstrated excellent CH_4_ sensing response in TiO_2_ nanofiber due to its impressive high surface area (1375.238 m^2^/g) and the presence of anatase phase (55%) [118]. Table 5 summarizes gas-sensing performances of 1D TiO_2_ nanostructures in recent publications.

Two-dimensional TiO_2_ nanostructures include nanosheets, nanoplates, and thin films, which possess specific advantages in gas-sensing applications compared to other low-dimensional nanostructures, as outlined below (I–IV). As a result, 2D TiO_2_ has been fabricated for gas-sensing applications.

I. The large surface area and high surface-to-volume ratio enable a greater number of atoms to interact with the atmosphere.
II. Higher mechanical stability.
III. With a relatively larger lateral size, 2D nanostructured materials provide a more conformal contact with electrodes compared to other low-dimensional nanostructures.
IV. The possibility of assembling into three-dimensional (3D) architectures.

**Figure 25:**
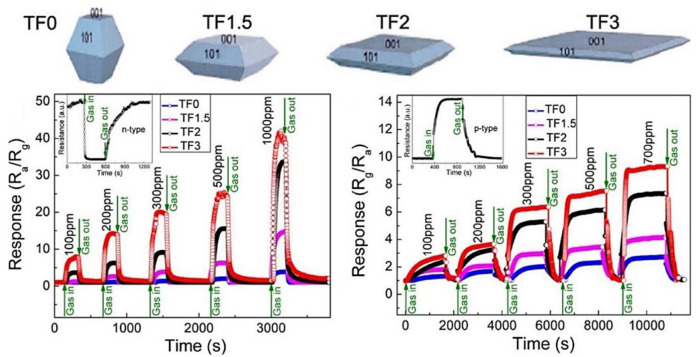
Both p-type and n-type sensing responses are revealed to be facet-dependent, demonstrating an obvious increase in response with increasing {001} percentage. Reprint from [140].

In this context, Liu and colleagues demonstrated an improvement in the sensor response to ethanol by increasing the exposure of the {001} crystal plane to ethanol in TiO_2_ nanosheets. This enhancement was attributed to the transformation of oxygen species from O^−^ to O^2−^ when increasing the exposure of the {001} crystal plane [138,139] (Figure 25). Furthermore, the proton transfer mechanism between ethanol molecules and adsorbed water molecules on the surface of TiO_2_ nanocrystals significantly influences the conductance type, altering it from the typical n-type to an unusual p-type behavior when operated at temperatures below 120 °C [140]. In line with this, Wang and colleagues demonstrated the impact of the {110} facet on the surface area on gas-sensing performance [141]. Additionally, the facilitation of gas diffusion and electron transfer in hierarchical structures led to a quick response. Anatase and rutile are the most common forms of TiO_2_, and numerous nanostructures have been created using each form. Combining these two forms improves ethanol-sensing properties, as shown by Zhang et al. [142]. The prepared A@R-TiO_2_ nanosheets, which are composed of anatase TiO_2_ (A-TiO_2_) nanosheets with rutile shell layers of 5 nm thickness, show four times better response to ethanol compared to A-TiO_2_ nanosheets when operated at 270 °C. This is due to the formation of TiO_2_ polymorphism junctions, which leads to electron transfer from the inner anatase cores to the outer rutile shells and increased concentration of active O^−^ species on the rutile shells, thus greatly improving the ethanol-sensing properties of the A@R-TiO_2_ nanosheets. Moreover, the distinct core–shell design featuring an enlarged surface area plays a role in elevating the resistance modulation and ethanol-sensing abilities of the A@R-TiO_2_ nanosheets. The increased surface area is also a factor leading to the improved resistance modulation and enhanced ethanol-sensing capabilities of the A@R-TiO_2_ nanosheets.

The ability to respond quickly and accurately is crucial for high-performance gas sensors, especially in applications such as breath acetone sensing. In this regard, Wanyin and colleagues reported the potential of detecting acetone in a mere 0.75 s with hierarchical TiO_2_ nanosheets [143]. Their study revealed that the surface area and the pore size of the sensing materials have a significant impact on performance. With a high specific surface area of 71.8 m^2^.g^−1^ and an average pore size of 21.3 nm, the plate-like TiO_2_ nanosheets demonstrated excellent acetone-sensing capabilities. The sensors also showed good response at high relative humidity levels, though a 25% drift in response was observed at 90% humidity due to the absorption of H_2_O molecules on the porous TiO_2_ surface. The gas-sensing properties of 2D TiO_2_ nanostructures are summarized in Table 6.

### 4.2. Advanced Techniques to Enhance Gas-Sensing Functionality

To improve gas-sensing capabilities, innovative methods are utilized, such as doping, decoration, carbon composites, and heterostructures. A brief overview of each of these techniques is mentioned below.

#### 4.2.1. Doping

Elemental doping is a process that adds elements to the crystal lattice of metal oxides (MOXs) through ion substitution. This results in changes to the original MOX properties and creates surface defects, reducing the grain size and increasing the active surface sites, which are more receptive to gas adsorption and reaction [151]. Additionally, the grain size and electronic band of nanomaterials are determined by the location of the defect sites and the host or doping ions, which affects the resistance of the sensing layer. Substituted atoms can also serve as active sites for gas adsorption. In other words, surface impurities, defects, and doping ions that generate adsorption sites can result in extrinsic electronic states [152,153,154].

In particular, TiO_2_ can be doped with metal (such as Co, Nb, Mn, Cu, and Ni) or non-metal dopants (such as C, F, and N) [155]. Studies have shown that metal doping can enhance the gas response and selectivity of TiO_2_ by altering phase transformation, surface potentials, chemical activity, amount of adsorbed oxygen ions, and regulating charge carrier concentration [156,157]. On the other hand, non-metal doping has been shown to alter the band gap of TiO_2_, support the formation of oxygen vacancies and active functional groups, enhance catalytic activity, and increase charge carrier concentration [158,159,160,161].

**Figure 26:**
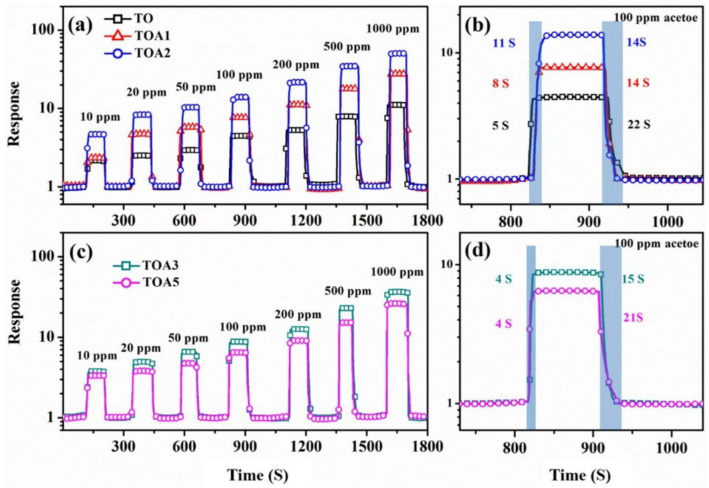
The dynamic response curves of sensors based on TO, TOA1, TOA2 (**a**), TOA3, and TOA5 (**c**) toward 10–1000 ppm acetone (TO at 315 °C, whereas TOA1, TOA2, TOA3, and TOA5 at 275 °C). The response and recovery times of TO, TOA1, TOA2 (**b**), TOA3, and TOA5 (**d**) are shown in the figure. Reprinted from [107].

Zhong and colleagues showed that adding 10% ZnO to TiO_2_ nanoparticles improved the sensitivity toward acetylene by 110% when compared to pure TiO_2_ NPs, and shortened the response time by 30% [162]. Similarly, the addition of 2 mol% Ag to TiO_2_ NPs led to a three-fold increase in the response to acetone, which was attributed to the electronic sensitization caused by the presence of Au [107] (Figure 26). Tshabalala and colleagues reported a six-fold increase in the sensor signal toward NH_3_ when 1.0 mol% Mn was added to TiO_2_ [108]. This improvement was a result of enhanced surface activity, higher concentration of Ti^3+^, and the presence of singly ionized oxygen vacancies. The doping of TiO_2_ nanoparticles with 20% Co^2+^ in cobalt nitrate resulted in a 7-fold increase in the response to 50 ppm NH_3_ compared to pure TiO_2_. The Co-doping introduced defects in the TiO_2_ lattice, reducing the band gap by 72% to 0.72 eV, thereby providing more active and reactive sites for TiO_2_ and leading to improved gas-sensing performance at room temperature [110]. Furthermore, doping TiO_2_ NPs with 15% Co_3_O_4_ (0.85 TiO_2_-0.15 Co_3_O_4_) resulted in a six-fold increase in the response to acetone (50 ppm) when operating at room temperature, and a three-fold increase under UV light exposure (360 nm). Loading with PAN (0.01 wt%) further improved the response by 2 times and maintained the sensor properties for 12 months [163]. On the other hand, doping 5% Cr into TiO_2_ NPs improved the stability of the sensors [164]. Carbon (C) doping, a non-metallic doping approach, has been well studied on TiO_2_ NPs for tuning their sensitivity to acetone [163]. In this case, C-doped TiO_2_ NPs (diameter 30 nm) showed an excellent response (5.4 times higher than pure TiO_2_) toward 100 ppm n-pentanol when operating at 170 °C, with the response increasing with the length of the carbon chain (CH_3_(CH_2_)_n−1_OH, n = 1–6) due to the increasing dissociative adsorption energy of CH_3_(CH_2_)_n-1_OH (n = 1–5). Additionally, Pd doping in CoTiO_3_/TiO_2_ (Pd-CTT) nanocomposite showed a 4-fold increase in response to benzene, compared to being undoped, at room temperature due to the formation of CoTiO_3_/TiO_2_ p–n heterojunction and the catalytic action of Pd [165].

Pristine TiO_2_ gas sensors face limitations in some gas-detection processes due to the high working temperatures required. Furthermore, high relative humidity also greatly affects the performance of metal oxide sensors. However, Na_0.23_TiO_2_/TiO_2_ NTs show exceptional NOx-sensing capabilities, even at 80% relative humidity [124]. These sensors have also demonstrated a seven-fold higher response compared to commercially available TiO_2_ particles. This improved performance can be attributed to the high concentrations of vacant (41.56%) and adsorbed (33.35%) oxygen, a large specific surface area (99.3 m^2^.g^−1^), and a small pore size (7 nm) in the material, as well as the formation of p–n heterojunctions between Na_0.23_TiO_2_/TiO_2_ and TiO_2_. Similarly, 0.1 M Co-doped TiO_2_ NTs show a 7.6-fold higher response to 500 ppm H_2_S compared to undoped TiO_2_ NTs [166].

Meanwhile, fluorine-doped TiO_2_ nanosheets demonstrate superior acetone-sensing functionality at room temperature compared to undoped TiO_2_ nanosheets due to the large specific surface area, formation of active functional groups, abundant oxygen vacancies, and high electron mobility [146].

#### 4.2.2. Loading/Decoration/Functionalization

**Figure 27:**
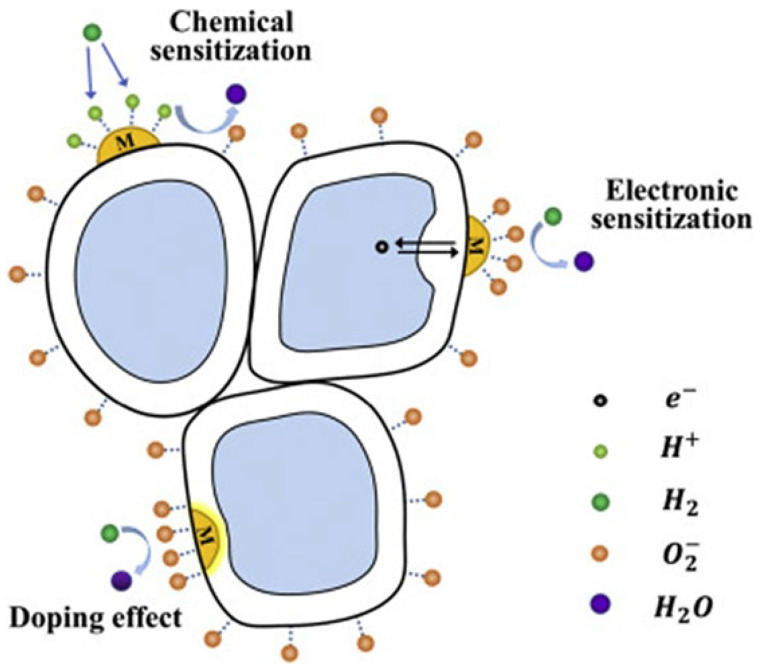
A schematic diagram showing the surface-sensitization effect (chemical sensitization and electron sensitization) and bulk doping of TiO_2_; here, M is labelled for metal, arrows represent the motion of election. Reprinted from [15].

Noble metal loading or surface decoration of MOXs is another method to improve their gas-sensing properties. This is achieved through chemical or electronic sensitization [16,167]. Chemical sensitization increases the ability of MOXs to adsorb oxygen molecules, which speeds up the reaction between the adsorbed oxygen and reducing gas molecules. Furthermore, the addition of a loading metal in some instances can result in a catalytic effect. This occurs when gas molecules are adsorbed and activated on the surface of the loading metal, leading to fast oxidation–reduction reactions with the adsorbed oxygen, thereby improving the sensing performance. Electron sensitization refers to the alteration of electron distribution in MOXs (TiO_2_) due to the different work functions between noble metals and MOXs. This results in the transfer of electrons between a noble metal and MOXs until they reach a balanced state, creating a new Fermi level [168]. This leads to a depletion of electrons on the surface of both the noble metal and MOXs, increasing the resistance at the interface between them in air and making the interface more responsive to resistance changes when a target gas is present (Figure 27) [15].

The sensitivity and selectivity of TiO_2_ NRs toward xylene are significantly improved when incorporating 5% of Ag due to the catalytic activity of Ag, which boosts the reactions between xylene molecules and adsorbed oxygen ions [120]. Mintcheva and colleagues showed that the selectivity of TiO_2_ nanoparticles toward ammonia, acetaldehyde, and benzene can be adjusted based on the degree of Au loading [104]. On the other hand, Lee and colleagues demonstrated excellent gas-sensing properties of TiO_2_ NPs decorated with NiO and PANI. The sensor showed a detection limit of 176.2 ppb under UV irradiation with a stability of up to six months, which were attributed to the creation of heterojunctions due to the loading of p-type materials, the catalytic effect of NiO upon its reaction with acetone molecules, the conversion of Ni ions resulting in additional oxygen adsorption, and the impact of UV illumination on PANI [111]. Chen et al. also showed the tunability of butane-sensing response based on the molar percentage of Pd on TiO_2_ NPs [102].

Xun et al. demonstrated a significant improvement in hydrogen (H_2_) sensing with SnO_2_-decorated TiO_2_ nanotubes. These nanotubes had a diameter of 3–8 nm and were placed within TiO_2_ nanotubes with an inner diameter of 100 nm, a wall thickness of 10–20 nm, and a length of 3–4 um. The H_2_ sensing was found to be 30 times higher compared to pristine TiO_2_ nanotubes, and the optimal operating temperature was decreased by 75 °C to 325 °C. Additionally, the response and recovery times were sped up by two times. These excellent results were attributed to the synergistic effect of hydrogen chemisorption, the large surface area, the nano-sized tube walls, the inter-wall connecting points, and the formation of heterojunctions at the interface between SnO_2_ and TiO_2_ [133]. Additionally, Chen et al. demonstrated a 1.89-fold increase in sensitivity and long-term stability in TiO_2_ nanofibers decorated with SnO NPs (50 nm in diameter) compared to pristine TiO_2_ NFs [132]. Furthermore, decorating TiO_2_ (rutile) nanorods (100 nm in diameter and 4.5 μm in length) with Pd (12.18 atomic%) resulted in a 35-fold increase in response to 1000 ppm H_2_ at 200 °C, compared to pristine TiO_2_ NRs. This enhancement was attributed to the spillover effect, the catalytic activity of the Pd particles (which enhanced the adsorption and desorption of O_2_), the electron sensitization, the formation of Pd hydride, and the enhancement of hydrogen absorption by Pd (acting as a hydrogen collector) [169].

Meanwhile, Alp et al. demonstrated that Ag loading on TiO_2_ NRs greatly improved the sensitivity and specificity for acetone. The catalytic effect of Ag on the surface of TiO_2_ NRs also decreased the operating temperature. The Ag-loaded TiO_2_ NR sensor showed a response for 65 ppb of acetone, while no response was observed from the pristine TiO_2_ NRs at the optimal operating temperature of 200 °C. Furthermore, the Ag-loaded TiO_2_ NRs demonstrated highly selective and sensitive acetone-sensing performance at 100 °C [131]. Cai et al. showed that by decorating TiO_2_ porous nanofibers with Co_3_O_4_ NPs, the sensitivity toward acetone could be significantly improved by over 9.3 times compared to pristine TiO_2_ [129]. Additionally, the response and recovery times were shortened by 11.4% and 13%, respectively. These enhancements were attributed to the formation of p–n heterojunction at the interface, the catalytic activity of Co_3_O_4_, and the grain boundaries between subgrains of TiO_2_ NFs. Meanwhile, Gakhar et al. reported a 93% increase in formaldehyde response through functionalizing TiO_2_ NTs with fullerene (C_60_) [127]. Additionally, C60-TiO_2_ nanotube composites, which featured an increased surface area and a highly reactive surface, led to a rapid response time of 4 s and a recovery time of 7 s. Zhao et al. showed that incorporating 1–3 layers of MoS_2_ nanoflakes into TiO_2_ nanotubes greatly enhanced the sensitivity toward ethanol, resulting in an 11-fold improvement compared to pure TiO_2_ nanotubes [170]. The improvement in the gas-sensing performance was credited to the synergistic interaction between MoS_2_ and TiO_2_, the creation of p–n heterojunctions, and the elevated surface area. Similarly, incorporating 6 mol% Nb_2_O_5_ into TiO_2_ NFs (Nb_2_O_5_-TiO_2_) enhanced the ethanol-sensing response by 2.79 times when compared to pure TiO_2_ NFs. This was because the incorporation of Nb_2_O_5_ led to a decrease in the average grain size and an increase in the specific surface area of Nb_2_O_5_-TiO_2_, compared to pure TiO_2_ nanofibers [119]. Additionally, the sensors were able to operate at a temperature that was 50 °C lower than that of pure TiO_2_ due to a decrease in resistance in the Nb_2_O_5_-TiO_2_ sensors. This reduction was attributed to the substitution of Ti^4+^ ions with Nb^5+^ ions and the formation of n–n junctions between Nb_2_O_5_ NPs and TiO_2_ NPs.

Wen et al. demonstrated that decorating TiO_2_ NSs with Ag NPs (5% mass ratio: AgNPs/TiO_2_ NSs) led to a high sensitivity toward NH_3_ when operating at room temperature. The Ag@TiO_2_ sensor was found to have 3 times higher response toward 50 ppm NH_3_ compared to pristine TiO_2_ NSs. The sensor demonstrated excellent performance even at 90% relative humidity (RH%). These exceptional gas-sensing properties, which were attributed to an increase in reactive sites, an acceleration of electron transfer onto the TiO_2_ surface, the spillover effect, and the synergistic effect due to Ag NP decoration, were further supported by [147].

**Figure 28:**
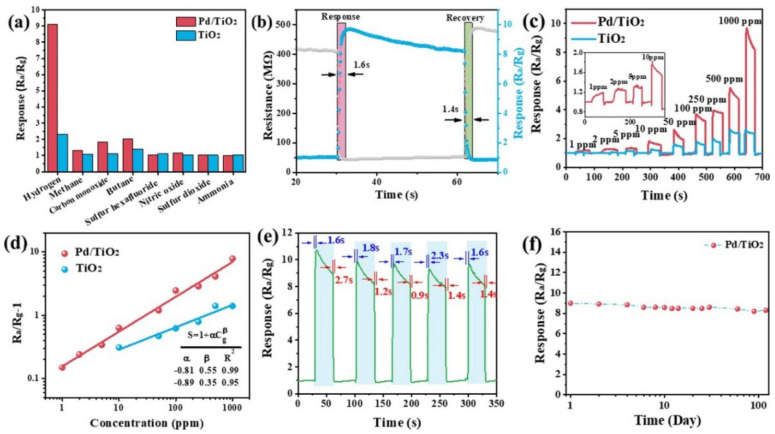
(**a**) Selectivity of TiO_2_ NS and Pd/TiO_2_ NS sensors toward different references gases (1000 ppm) when operating at 230 °C; (**b**) response/recovery curve of the sensor based on Pd/TiO_2_ NS toward 1000 ppm H_2_ when operating at 230 °C; (**c**) dynamic test curves of the sensors based on TiO_2_ NS and Pd/TiO_2_ NS when exposed to H_2_ with increasing concentration from 1 ppm to 1000 ppm and operating at 230 °C; (**d**) relationships between R_a_/R_g_-1 and H_2_ concentration of Pd/TiO_2_ NS sensors when operating at 230 °C; and (**e**) reproducibility and (**f**) long-term stability of the sensor based on Pd/TiO_2_ NS toward 1000 ppm H_2_ when operating at 230 °C. Reprinted from [148].

Pd nanocrystal-loaded (1.5 wt%) TiO_2_ porous nanotubes (diameter 3–8 nm) showed fast response and recovery times (1.6 s and 1.4 s, respectively) toward hydrogen (H_2_) when operating at 230 °C (Figure 28) [148]. The high surface area (90.33 m^2^.g^−1^), the open pores, the short channel length, and the formation of p–n heterojunction (PdO/TiO_2_) were all contributing factors to the excellent H_2_-sensing properties. Furthermore, this study revealed a higher potential of chemical adsorption of H_2_ and oxygen (absorption energy of −1.22 eV and −2.37 eV, respectively) on the PdO/TiO_2_ surface compared to TiO_2_ (absorption energy of −1.04 eV and −1.91 eV, respectively), making it easier for H_2_ to react with the chemisorbed oxygen on the PdO/TiO_2_ surface.

#### 4.2.3. Heterojunctions/Heterostructures

**Figure 29:**
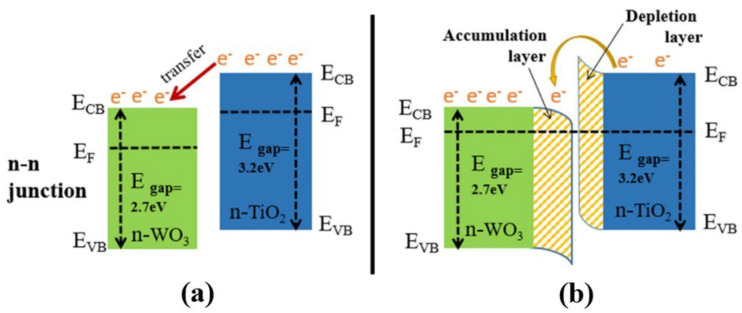
The formation of an n–n heterojunction at the interface between TiO_2_ and WO_3_: (**a**) the directional transfer of electrons; and (**b**) the formation of an electron-depletion layer in TiO_2_ and an electron-accumulation layer in WO_3_. Reprinted from [172].

TiO_2_ is generally an n-type semiconducting material with a relatively high level of gas sensitivity compared to p-type materials. However, studies on p-type materials, such as Co_3_O_4_ and Bi_2_O_3_, have shown that they offer exceptional gas-sensing capabilities at low operating temperatures, with enhanced selectivity and stability [171]. The combination of different metals in heterojunctions, referred to as n–n, n–p, and p–p heterojunctions (Figure 29) [172], enhances selectivity and other important gas-sensing parameters. When two materials form a heterostructure connection, their Fermi levels (EF) align, causing electrons with higher energy to flow to unoccupied lower-energy states until a balance is reached. This creates a depleted zone of charge carriers at the interface (depletion region). The difference in the original Fermi levels between the materials produces a potential energy barrier at the interface, leading to higher resistance at the junction in air and heightened sensitivity toward the presence of analyte gas.

**Figure 30:**
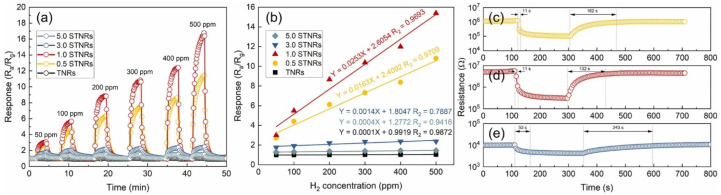
(**a**) Response–recovery curves of the sensors based on TNRs and STNRs toward H_2_ with various concentrations at 100 °C. (**b**) Relationships between the response and H_2_ concentration for the TNRs and STNRs. (**c**–**e**) Response/recovery times of the 0.5 STNRs, 1.0 STNRs, and 3.0 STNRs toward 500 ppm H_2_, respectively. Reprinted from [115].

The ability to change between n-type and p-type semiconductor characteristics was demonstrated in hierarchical NiO (111)/TiO_2_ (002) junction by adjusting the thickness of NiO NSs. For instance, NiO NSs with a 367.1 nm pore size and a 20 nm thickness on TiO_2_ NRs exhibited n-type behavior during hydrogen sensing, while a pore size of 232.7 nm and a thickness of 1585 nm resulted in p-type semiconducting characteristics [173]. Furthermore, TiO_2_/α-Fe_2_O_3_ n–n junction arrays, with α-Fe_2_O_3_ branches adorning TiO_2_ NRs, showed a significant increase in response to 100 ppm acetone at 225 °C compared to pure TiO_2_ NRs. The increase in response was attributed to the increased surface area (10.49–16.78 m^2^.g^−1^) due to the α-Fe_2_O_3_ branches, the synergistic effect of oxygen adsorption and electron transport, the additional pathway for electron transfer, and the formation of an n–n junction [135]. Alev showed considerable enhancement in hydrogen sensing at 200 °C in a p-Co_3_O_4_-n-TiO_2_ nanotube structure compared to a pristine TiO_2_ nanotube. This enhancement was due to the catalytic nature of Co_3_O_4_ and the creation of a p–n junction at the boundary between Co_3_O_4_ and TiO_2_, altering the electrical properties of the device [174]. Consequently, the CuO thin film/TiO_2_ nanotube structure displayed enhanced performance in hydrogen sensing, while boasting a low operating temperature, low detection limit, and high sensor response compared to both standalone TiO_2_ NTs and CuO thin film. The heterostructure not only improved hydrogen sensing but also significantly diminished the response to volatile organic compounds and nitrogen dioxide. The improved sensing properties were credited to the heterojunction created between the CuO thin film and TiO_2_ nanotubes [123]. Li et al. showed that SnO_2_@TiO_2_ heterostructured NRs had quicker response time (11 s) and recovery time (132 s), as well as superior stability and specificity in detecting hydrogen (H_2_) at 100 °C in comparison to pure TiO_2_ nanorods. (Figure 30) [115]. The enhancement was credited to the enlargement of NR diameter from 160 nm to 190 nm through the dispersal of 7 nm SnO_2_ NPs on the cross-linked areas of neighboring TiO_2_ nanorods, leading to the modulation of the conduction path, potential barrier, and chemical/electron sensitization. Similarly, Wang and colleagues showed an 8-fold increase in response and quick response/recovery time for detecting acetone at 300 °C in SnO_2_ NPs-TiO_2_ NB structures compared to TiO_2_ NBs [175]. The elevated sensitivity of the SnO_2_/TiO_2_ sensor was a result of the large surface area provided by SnO_2_ NPs, and the fast response/recovery time was attributed to the fast carrier transport speed along the one-dimensional axis of the TiO_2_ NBs.

Metal oxide incorporation is another technique to enhance gas-sensing properties. For instance, embedding SnO_2_ clusters into TiO_2_ nanosheets forms heterojunctions, as well as changing the reactive active sites and the surface area [144]. Haidry et al. showed an 8.5-fold enhancement toward 100 ppm ethanol in Ag/TiO_2_ heterojunction compared to that of TiO_2_ nanosheets (100 nm) [150]. Additionally, the sensors demonstrated fast repose/recovery times of (9/10 s), which were ascribed to the strongly coupled Ag/TiO_2_ nanocomposites, with metallic Ag NPs being supported or partly encapsulated by the single-crystal TiO_2_ nanosheets (≤10 nm) through an intimate contact interface.

### 4.3. Carbon Hybrids

The exceptional and distinctive characteristics of carbon-based materials, such as carbon nanotubes, graphene, and graphite, provide a major advantage in the creation of advanced composites. The effectiveness of these materials as a matrix component is largely determined by the interaction between the matrix and other materials. Typically, the integration of C-based materials into MOX structures leads to the conversion from n-type to p-type or the formation of a p–n junction, making active sites available for gas adsorption and creating the desired depletion layer [176].

Combining TiO_2_ with carbon materials, such as graphene and CNTs, is a promising way to improve the gas-sensing properties of TiO_2_-assisted sensors. [177]. Accordingly, combining TiO_2_ with carbon materials, such as graphene and carbon nanotubes, has been found to be an effective method for enhancing the gas-sensing properties of TiO_2_-assisted sensors [178,179]. Additionally, the fabrication of a hybrid structure based on p-type reduced graphene oxide (RGO) and n-type metal oxide leads to the formation of a p–n heterojunction, which increases the Schottky barrier and enhances the gas-sensing response of the material [122]. Preparing hybrids has several benefits for gas sensors, including higher charge transfer rates [180,181], improved charge carrier transport [182,183], and ability to construct heterojunctions [184].

Ramos and colleagues showed that the response of carbon gel–TiO_2_ nanocomposites increased with the weight percentage of TiO_2_ in the composites. The highest response was achieved with 80 wt% of TiO_2_ NPs (20–30 nm, rutile) in the composites for detecting NH_3_ at room temperature. Additionally, the response of the sensors was improved by UV illumination [105]. As a result, the TiO_2_/graphene hybrids demonstrated two times higher response to NO_2_ compared to pure TiO_2_ nanoparticles when operating at room temperature with the illumination of 180 mW UV light. This enhancement was achieved due to the well-connected graphene sheets that promote electronic transport, thus increasing the carrier generation and photo-dissociation of the target gas along with UV irradiation [106].

Furthermore, the combination of C_60_ with TiO_2_ NPs (150 nm) resulted in improved response and quick response time toward formaldehyde detection compared to pure TiO_2_ NPs (18 nm) and C60-functionalized TiO_2_ NPs. The improvement in sensing performance was attributed to the increased reactivity of C60 and the synergistic effect of both C60 and TiO_2_ nanoparticles, as well as the fast electron exchange between the ambient conditions and TiO_2_ [122].

Combining conductive polymers with MOXs is a feasible solution to lower the operating temperature. For example, PANI/TiO_2_ NR composite demonstrated remarkable detection of dimethylamine (DMA) with a response of 6.36 for 30 ppm at room temperature, which was significantly better than the response of 1.47 for pure TiO_2_ NR arrays [136]. This was attributed to the distinctive morphology structure and the formation of p–n heterojunctions at the interface between the PANI and TiO_2_ nanorod arrays, which enhanced the gas-sensing capability. Similarly, Safe et al. reported that the response and recovery time for NH_3_ sensing were improved by five times in TiO_2_ (30% rutile and 70% anatase)/PANI core–shell (thickness of 20 nm) nanofibers (diameter of 360 nm) with UV irradiation compared to those without UV irradiation [137]. Moreover, the sensor exhibited p-type gas-sensing properties with remarkable sensitivity, even at a humidity of 99 RH%. This was attributed to the conducting dominance of the p-type PANI shell instead of the n-type TiO_2_.

**Figure 31:**
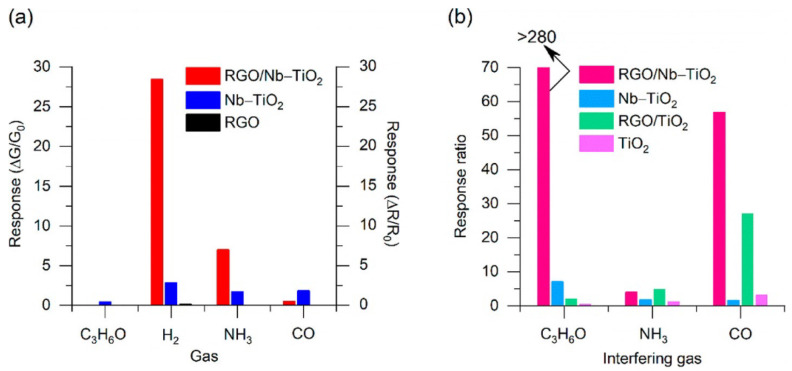
(**a**) Response of RGO/Nb–TiO_2_ (prepared using 9 ng/mm^2^ GO, optimal composition), Nb–TiO_2_ NTs, and RGO (prepared with 9 ng/mm2 GO) toward C_3_H_6_O (120 ppm), H_2_ (120 ppm), CO (120 ppm), and NH_3_ (30 ppm) at their optimal working temperature (200 °C). (**b**) Response ratio calculated as the response toward H_2_ (120 ppm) with respect to C_3_H_6_O (120 ppm), CO (120 ppm), and NH_3_ (30 ppm) for RGO/Nb–TiO_2_ (prepared using 9 ng/mm^2^ GO), Nb–TiO_2_, RGO/TiO_2_ (prepared with 4.5 ng/mm^2^ GO, the optimal composition of RGO/TiO_2_), and pristine TiO_2_ samples at an operating temperature of 200 °C. The RGO/Nb–TiO_2_ hybrid material (prepared with 9 ng/mm^2^ GO) is not sensitive toward C_3_H_6_O at the optimal operating temperature, so we can only provide a lower bound for the response ratio: >280 means that the response of RGO/Nb–TiO_2_ (prepared using 9 ng/mm^2^ GO) toward C_3_H_6_O is 0.1 when calculating the H_2_/C_3_H_6_O response ratio. Reprinted from [122].

Additionally, the incorporation of Nb into TiO_2_ nanotube hybrid (reduced graphene oxide, RGO) and the degree of reduction and concentration of RGO have a significant impact on enhancing gas-sensing selectivity due to the catalytic role of RGO (Figure 31) [122]. Besides secondary hybrids, ternary hybrids are also gaining attention as gas-sensing capability can be further improved by the addition of a third element. For instance, Pd/RGO/TiO_2_ NTs ternary hybrid utilized the individual contributions of TiO_2_ nanotubes (density of 50–62 um^−2^, primary sensing element), RGO (high-mobility distributed connector), and Pd nanoparticles (diameter of 6–16 nm, enhance sensing performance through catalytic effect) in a synergistic manner. The developed ternary hybrid structure demonstrated outstanding sensing performance in terms of response magnitude (96% toward 1000 ppm methanol) and response/recovery times (11 s/15 s) [134].

Consequently, Wand and colleagues showed that TiO_2_ nanosheets with an exposed {001} facet has excellent acetone-sensing performances compared to TiO_2_ nanorods with an exposed {110} facet because of the high-energy {001} [185,186] crystal facets of TiO_2_ and TiO_2/_SiC heterojunctions [183]. Murali et al. found a 4.8-fold enhancement in the response toward 100 ppm NO in TiO_2_@NGQDs compared to pristine TiO_2_ nanoplates (length of 42 nm and thickness of 4.2 nm) [149]. In this case, decorating the {001} facets of the TiO_2_ nanoplates, which is highly reactive for the adsorption of active oxygen species, with nitrogen-doped graphene quantum dots (NGQDs, 2 nm thick layer on TiO_2_ NPLs) dramatically enhanced the efficiency of gas and carrier exchange, the charge carrier separation and transportation, and oxygen vacancies, which eventually improved the sensing performance. Furthermore, the sensors demonstrated 31.1% response toward NO when UV irradiation was employed. However, the best response was 223% toward NO when operating at 250 °C.

## 5. Future Perspectives and Challenges

The synthesis of TiO_2_ nanostructures and their application in gas sensing have gained significant attention in recent years, and they hold promising future prospects as sustainable materials for environmental monitoring and pollution control. However, along with the opportunities, there are several challenges that need to be addressed for the successful implementation of TiO_2_ nanostructures in gas-sensing applications.

One of the key prospects in the future of TiO_2_ nanostructures for gas sensing is their sustainable synthesis. With increasing concern about environmental pollution and the need for sustainable technologies, the synthesis of TiO_2_ nanostructures using green and eco-friendly methods is gaining importance. Researchers are exploring various green synthesis approaches, such as sol–gel methods using renewable resources, hydrothermal methods using biodegradable templates, and microwave-assisted methods using green solvents. These sustainable synthesis approaches aim to reduce the environmental impact of TiO_2_ nanostructure synthesis, minimize the use of hazardous chemicals, and improve the energy efficiency of the synthesis processes.

Another prospect is the potential for TiO_2_ nanostructures to be integrated into gas-sensing devices for environmental monitoring and industrial applications. TiO_2_ nanostructures possess excellent gas-sensing properties, including high sensitivity, fast response, and low power consumption. They can detect a wide range of gases, such as volatile organic compounds (VOCs), nitrogen oxides (NO_x_), carbon monoxide (CO), and hydrogen (H_2_), making them suitable for various gas-sensing applications, including indoor air quality monitoring, automotive emission control, and industrial process monitoring. In the future, we can expect to see more advanced gas-sensing devices incorporating TiO_2_ nanostructures that provide real-time, accurate, and reliable gas detection for environmental and industrial purposes.

However, there are also challenges that need to be addressed in the future development of TiO_2_ nanostructures for gas sensing. One challenge is the improvement of the selectivity and stability of TiO_2_-based gas sensors. TiO_2_ nanostructures can exhibit cross-sensitivity to different gases, leading to false readings and reduced specificity. Additionally, the long-term stability and durability of TiO_2_-based gas sensors can be affected by various factors, such as temperature, humidity, and aging, which can impact their reliability and performance over time. Developing strategies to enhance the selectivity and stability of TiO_2_-based gas sensors is crucial for their practical applications in real-world environments.

Another challenge is the scalability and cost-effectiveness of TiO_2_ nanostructure synthesis. While there has been significant progress in the synthesis of TiO_2_ nanostructures, scaling up these synthesis methods to meet the demands of large-scale production can be challenging. Cost-effective synthesis methods that can produce TiO_2_ nanostructures in large quantities while maintaining their high quality and properties need to be developed to enable their widespread commercialization and industrial applications.

Furthermore, the potential environmental and health impacts of TiO_2_ nanostructures need to be thoroughly investigated in the future. As TiO_2_ nanostructures become more widely used in gas-sensing applications, it is important to assess their potential impacts on the environment and human health. Studies on their toxicity, biocompatibility, and environmental fate will be essential to ensure their safe and responsible use in various applications.

## 6. Conclusions

Recent research has shown considerable interest in creating high-performance gas-sensing devices using MOX nanostructures, especially TiO_2_. However, the use of 2D TiO_2_ nanostructures in gas sensing is not widespread, and only a limited number of studies have been reported in recent years. Unfortunately, many individuals still resort to costly and environmentally hazardous synthesis methods, despite the availability of sustainable alternatives.

In recent years, the focus has been on enhancing the sensing performance of TiO_2_ nanostructures through metal nanoparticle doping, decoration, or MOX functionalization. These modifications result in composites or heterostructures that exhibit improved sensitivity and selectivity; lower operating temperatures; and faster response and recovery times. These improvements are attributed to p–n junction formation and catalytic and synergetic effects from newly engineered composite structures.

In conclusion, TiO_2_ nanostructures have a promising future in gas-sensing applications due to their sustainable synthesis methods, excellent gas-sensing properties, and potential for integration into environmental monitoring and industrial devices. However, several challenges, such as improving selectivity, stability, scalability, and cost-effectiveness of synthesis, as well as addressing potential environmental and health impacts, need to be overcome for their widespread adoption. Future research and development efforts in these areas will play a critical role in advancing the field of TiO_2_ nanostructures for sustainable gas-sensing technologies. Novel engineering techniques, such as doping, functionalizing, and preparing heterostructures, are being utilized to enhance gas-sensing performance. It is important to use eco-friendly solvents and green co-precipitation in hydro/solvothermal processes for environmental sustainability.

## Figures and Tables

**Table 1:** A summary of synthesized TiO_2_ nanostructures using the sol–gel method.

Chemical Compounds	Synthesis Conditions	Structural and Morphological Properties	Ref.
Titanium isopropoxide (TTIP) Deionized water (DI) Isopropanol	Stirring: TTIP + isopropanol—Time: 30 min Stirring: TTIP + isopropanol + DI water—Time: 2 h Ultrasonic treatment: Temp: 80 °C—Time: 1 h Aging temp: RT—Time: 22 h Washing: DI water and ethanol Drying temp: 70 °C—Time: 2 h Calcination—300–800 °C	Phase: anatase + rutile Crystallite size: increases with increase in calcination temperate from 8.45 to 38.55 nm Calcination (300 °C) Morphology: nanoparticles with roughly spherical and spongy shape Particle size: ~35 nm Calcination (800 °C) Morphology: non-uniform particles	[32]
Citric acid Ethanol TTIP Ethylene glycol (EG)	Stirring: citric acid + ethanol + TTIP Temp: RT—Time: 1 h Doping: NbOEt_5_ and La (NO_3_) 3·6H_2_O added to the solution Stirring temp: RT—Time: 1 h Stirring: solution + EG Calcination time: 2 h—Temp: 400 °C	Phase: anatase Morphology: semi-spherical nanoparticles Crystallite size: ~30 nm	[38]
Titanium chloride (TiCl_4_) *Malva parviflora* extract (MPE) Deionized water	Stirring: TiCl_4_ + MPE + DI water Time: 3 h Doping: Thiourea added to have S^2−^ Aging time: 24 h—Temp: 60 °C Calcination temp: 400 °C—Time: 3 h	Phase: Anatase related to TiO_2_ Morphology: S-doped TiO_2_ nanoparticles Particle size: 20.3 nm	[39]
TTIP Glacial acetic acid Double-distilled water	Stirring: TTIP + glacial acetic acid + double-distilled water—Time: 5 h Ultrasonic treatment: Time 30 min Aging time: 24 h in dark Thermal treatment: Temp: 80 °C—Time: 10 h Drying temp: 100 °C Calcination temp: 600 °C—Time: 5 h	Phase: anatase Crystallite size: ~10.88 nm Morphology: spherical and rod Particle size: ~121 nm	[40]
*Phyllanthus niruri* leaf extract Distilled water TTIP	Plant Stirring: *Phyllanthus niruri* leaf + distilled water Time: 2 h—Temp: 50 °C TiO_2_ preparation Stirring: TTIP + plant extract Time: 4 h—Temp: RT Centrifuging time: 20 min Drying temp: 80 °C—Time: 24 h Calcination temp: 600 °C—Time: 3 h	Phase: anatase Crystallite size: ~20.90 nm Morphology: nanoparticles	[41]
Plumeria leaves TTIP Hexane Ethanol	Stirring: TTIP + hexane + plant extract Washing: ethanol Drying temp: 100 °C—120 °C Calcination temp: 500 °C—Time: 4 h	Phase: anatase Morphology: nanoparticles Particle size: 77.6 nm	[42]
Te (VI) acid Ti (IV) butoxide EG	Stirring A: Ti (IV) butoxide + EG Stirring B: Te (VI) acid + EG Stirring: A + B pH: 7.5–8 Heat treatment temp: 200 °C Calcination temp: 300 °C to 700 °C—Time: 2 h	Phase: anatase Crystallite size: ~60 nm Morphology: spherical nanoparticles Particle size: 50 to 100 nm	[43]
TBOT Ethanol	Stirring: TBOT + ethanol Time: 2 h—Temp: RT Washing: ethanol Drying time: 10 h—Temp: 60 °C Calcination temp: 200 °C—Time: 2 h	Phase: anatase Morphology: spherical particles Particle size: 30–40 nm	[44]
TTIP Ethanol Cobalt acetate Cadmium acetate Urea	N-Co-Cd-doped TiO_2_ nanocomposite Stirring: TTIP + ethanol—Time: 30 min Doping: 1% Co, Cd, and N Drying temp: 100 °C—Time: 24 h Calcination temp: 550 °C—Time: 4 h	Phase: anatase + β-Cadmium nitrate-cobalt titanium + titanium nitride Morphology: mesoporous nanocomposite	[45]
TTIP Isopropanol CuCl_2_.2H_2_O	Stirring solution 1: TTIP + isopropanol Temp: RT—Time: 2 h Stirring solution 2: CuCl_2_.2H_2_O + isopropanol Doping: Solution 1 added to solution 2 Calcination time: 2 h—Temp: 400–500–600 °C	Doping concentration: 5% Calcination temp: 400 °C Phase: anatase Crystallite size: 4.63 nm Calcination temp: 500 °C Phase: rutile Crystallite size: 8.7 nm	[46]
TiCl_4_ Distilled water Ethanol	Stirring: TiCl_4_ + distilled water + ethanol Time: 30 min—Temp: RT pH: from 1 to 10 Aging time: 12 h Washing: distilled water Drying temp: 120 °C—Time: 1 h Calcination temp: 500, 600, 700, and 800 °C Time: 4 h	Phase: anatase Morphology: non-uniform cluster of TiO_2_	[47]

**Table 2:** A summary of synthesized TiO_2_ nanostructures using green methods.

Plant	Synthesis Conditions	Structural and Morphological Properties	Ref.
Leaf of *Trigonella foenum*	Plant extract Stirring: leaves + distilled water Time: 20 min TiO_2_ preparation Stirring: titanium oxysulphate + plant extract + NaOH Calcination temp: 700 °C—Time: 3 h	Phase: anatase Morphology: spherical NPs Particle size: ≈25 nm	[59]
*Citrus limetta* leaves	Plant extract Boiling: leaves + distilled water Temp: 100 °C—Time: 15 min TiO_2_ preparation Stirring: titanium butoxide + distilled water + citrus limetta extract Time: 5 h Aging time: 24 h—Temp: RT Drying temp: 150 °C—Time: 2 h Calcination temp: 550 °C—Time: 2 h	Phase: anatase Gain size: 45 nm Morphology: NPs with spherical shape Particle size: 80–100 nm	[60]
Leaf of piper betel	Plant extract Stirring: leaves + distilled water Time: 30 min—Temp: 50 °C TiO_2_ preparation Stirring: TTIP + distilled water + plant extract Time: 3 h—Temp: 70 °C Calcination temp: 400 °C—Time: 3 h	Phase: anatase Morphology: rounded shape and formed in clusters Particle size: 6.4 nm	[67]
Leaf of *Ocimum tenuiflorum*		Phase: anatase Morphology: rounded shape and formed in clusters Particle size: 7 nm	
Leaf of *Moringa oleifera*		Phase: anatase Morphology: rounded shape and formed in clusters, with many aggregated particles Particle size: 6.6 nm	
Leaf of *Coriandrum sativum*		Phase: anatase Morphology: rounded shape and formed in clusters Particle size: 6.8 nm	
*Syzygium cumini* (jamun) leaves	Plant extract stirring: leaf powder + distilled water Temp: 80 °C—Time: 60 min TiO_2_ preparation Stirring: TTIP + Syzygium cumini extract Temp: RT—Time: 8 h Drying temp: 100 °C—Time: one night Calcination temp: 570 °C—Time: 3 h	Phase: anatase Crystallite size: 10 nm Morphology: Spherical Particle size: ≈22 nm	[68]
*Jatropha curcas* leaves	Plant extract Stirring: leaves + DD water Temp: 80 °C—Time: 40 min TiO_2_ preparation Stirring: TiCl_4_ + leaf extract Temp: RT—Time: 2 h Ammonia was added to the solution Washing: ethyl alcohol Calcination temp: 450 °C—Time: 3 h	Phase: anatase Crystallite size: ≈ 13 nm Surface area: 27.038 m^2^/g Pore size diameter: 19.1 nm Pore volume: 0.1291 cm^3^/g Morphology: spherical	[69]
*Ledebouria revoluta* (African hyacinth)	Plant extract Stirring: leaves + ethanol Time: 20 min TiO_2_ preparation Stirring: titanium dioxide + distilled water + extract Temp: 50 °C—Time: 4 h	Phase: tetragonal Crystallite size: 47 nm Morphology: spherical	[70]
Leaves of *Ocimum sanctum*	Plant extract Stirring: leaves + distilled water Time: 10 min—Temp: 80 °C—Stored at 4 °C TiO_2_ preparation Stirring: TiO_2_ + plant extract Temp: RT—Time: 6 h	Phase: anatase Morphology: spherical and polygonal Particle size: 75–123 nm	[71]

**Table 4:** Gas-sensing functionality of 0D TiO_2_ nanostructures.

Material	Method	Temp (°C)	Gas Conc. (ppm)	Resp.	Resp./Reco. Time (s)	LOD (ppb)	Stab. Repe.	Ref.
TiO_2_	hydrothermal	270	500 C_3_H_6_O	9.19 ^a^	10/9	500	10 days and 4 cycles	[101]
7.5 mol% Pd-TiO_2_	hydrothermal	400	3000 C_4_H_10_	39.63 ^a^	13/8	N/A	30 days and 7 cycles	[102]
C_60_–TiO_2_	sol–gel	150	100 CH_2_O	1.20 ^b^	12/331	1000	N/A 3 cycles	[103]
Au@TiO_2_	laser-irradiated	29	200 NH_3_	64 ^a^	28/24	5000	90 days N/A	[104]
			200 C_2_H_4_O	115 ^a^	59/78	40,000		
			200 C_6_H_6_	19.76 ^a^	39/26	50,000		
Carbon gel–TiO_2_	sol–gel	RT	100 NH_3_	18.8 ^b^	50/70	1550	150 days and 5 cycles	[105]
TiO_2_/GO	sol–gel	RT/UV	1.75 NO_2_	1.17 ^a^	35/90	50	N/A N/A	[106]
Au@TiO_2_	hydrothermal	275	100 C_3_H_6_O	13.9 ^a^	11/14	N/A	30 days N/A	[107]
Mn@TiO_2_	hydrothermal	RT	20 NH_3_	127.39 ^a^	21/24	440	N/A N/A	[108]
C-TiO_2_	hydrothermal	170	100 C_5_H_12_O	11.12 ^a^	100/675	500	120 days and 10 cycles	[109]
Co@TiO_2_	wet chemical	RT	50 NH_3_	14 ^a^	25/48	1000	30 days N/A	[110]
0.01 wt% PANI- 0.1 NiO–0.9 TiO_2_	sol–gel	25	50 C_3_H_6_O	10.3 ^b^	150/290	176.2	6 months and 4 cycles	[111]

^a^—R_a_/R_g_, ^b^—(R_a_-R_g_)/R_a_.

**Table 5:** Gas-sensing functionality of 1D TiO_2_ nanostructures.

Material	Method	Temp. (°C)	Gas Conc. (ppm)	Resp.	Resp./Reco. Time (s)	LOD (ppb)	Stab. Repe.	Ref.
TiO_2_ NWs	Hydrothermal	RT	100 NO_2_	3.1 ^a^	10/19	N/A	20 days N/A	[81]
SnO_2_@TiO_2_ NRs	Hydrothermal	100	500 H_2_	15.4 ^a^	11/132	N/A	N/A 4 cycles	[115]
TiO_2_ NWs	Sol−gel dip coating	340	100 C_2_H_5_OH	9.2 ^a^	13/46	N/A	50 days and 5 cycles	[117]
TiO_2_ NFs	Hydrothermal	23	60 CH_4_	57 ^a^	66/162	250	N/A N/A	[118]
6 mol% Nb_2_O_5_-TiO_2_ NFs	Electrospinning	250	500 C_2_H_5_OH	21.6 ^a^	N/A	41	20 days N/A	[119]
0.5 at% Ag/TiO_2_ NRs	Facile hydrothermal	375	100 C_8_H_10_	6.5 ^a^	5/2	N/A	15 days and 3 cycles	[120]
TiO_2_ NWs	Electrospinning	400	500 C_2_H_5_OH	11.9 ^a^	8/1	N/A	N/A N/A	[121]
Nb @ TiO_2_ NTs	Anodic oxidation	200	120 H_2_	28.4 ^b^	930/300	N/A	N/A N/A	[122]
CuO-TiO_2_ NTs	Anodization	200	1000 H_2_	2 ^d^	444/408	N/A	N/A N/A	[123]
Na_0.23_TiO_2_/TiO_2_ NTs	Hydrothermal	RT	10 NO_X_	5.9 ^a^	7/N/A	1.8	60 days and 4 cycles	[124]
C(60) 0.02 w% @ TiO_2_ NTs	Anodization	150	100 CH_2_O	0.9 ^a^	4/7	100	23 days and 3 cycles	[125]
TiO_2_ NTs	Anodization	300	50 H_2_S	26 ^a^	22/6	N/A	70 days N/A	[126]
TiO_2_ NRs	Hydrothermal	RT	60 CH_4_	6028 ^a^	<70/<70	329	30 days and 3 cycles	[127]
TiO_2_ NRs	Hydrothermal	100	150 H_2_	0.53%^b^	85/620	N/A	N/A N/A	[128]
Co_3_O_4_@TiO_2_ porous NFs	Hydrothermal	250	100 C_3_H_6_O	71.88 ^a^	122/351	N/A	30 days and 20 cycles	[129]
TiO_2_ NRs	Hydrothermal	320	100 C_3_H_6_O	12.3 ^a^	3/292	N/A	25 days and 6 cycles	[130]
Ag@TiO_2_ NRs	Hydrothermal	200	3.8 C_3_H_6_O	7.31 ^d^	1380/2280	65	N/A N/A	[131]
SnO_2_@TiO_2_ NFs	Facile electrospinning	240	100 C_2_H_5_OH	9.53 ^a^	8/10	N/A	40 days N/A	[132]
SnO_2_@TiO_2_ NTs	Anodization	250	1000 H_2_	1410 ^a^	2/~400	120,000	9 days and 5 cycles	[133]
Pd/RGO/TiO_2_ NTs	Anodization	50	700 CH_3_OH	0.96 ^b^	11/15	N/A	6 days and 3 cycles	[134]
TiO_2_/α-Fe_2_O_3_ NRs	Hydrothermal	225	100 C_3_H_6_O	21.9 ^b^	12/9	36	30 days and 5 cycles	[135]
PANI/TiO_2_ NRs	Hydrothermal	RT	30 C_4_H_9_NO	6.36 ^a^	100/349	50	30 days and 5 cycles	[136]
PANI/TiO_2_ NFs	Electrospining	RT/UV assisted	1 NH_3_	1.09 ^c^	63/37	50	60 days and 4 cycles	[137]

^a^—R_a_/R_b_, ^b^—(R_a_-R_g_)/R_a_, ^c^—(R_g_-R_a_)/R_a_, ^d^—(I_g_-I_a_)/I_a_.

**Table 6:** Gas-sensing functionality of 2D TiO_2_ nanostructures.

Material	Method	Temp. (°C)	Gas Conc. (ppm)	Resp.	Resp./Reco. Time (s)	LOD (ppb)	Stab. Repe.	Ref.
TiO_2_ (NSs)	Hydrothermal	310	500 C_2_H_5_OH	25.4 ^a^	N/A/N/A	1.4	N/A N/A	[139]
Core–shell TiO_2_ (NSs)	Hydrothermal	270	500 C_2_H_5_OH	43.9 ^a^	N/A/N/A	5.7	30 days N/A	[142]
Porous TiO_2_ (NSs)	Hydrothermal	400	200 C_3_H_6_O	21.6 ^a^	0.75/0.5	500	150 days N/A	[143]
SnO_2_/TiO_2_ (NSs)	Solvothermal	300	100 C_6_H_15_N	19.8 ^a^	8/N/A	N/A	60 days N/A	[144]
Hierarchical TiO_2_ (NSs)	Hydrothermal	25	50 NH_3_	122.6 ^a^	53/23	500	N/A 5 cycles	[145]
Fluorine-doped TiO_2_ (NSs)	Hydrothermal	25	100 C_3_H_6_O	5.95 ^b^	160/220	N/A	35 days and 5 cycles	[146]
Au5%@TiO_2_ (NSs_)_	Facile and in situ process	25	50 NH_3_	71.8 ^a^	38/168	5000	16 days and 5 cycles	[147]
Porous Pd@TiO_2_ (NSs)	Template assisted	230	1000 H_2_	9 ^a^	1.6/1.4	1000	100 days and 5 cycles	[148]
TiO_2_@NGQDs (NPTs)	Hydrothermal	250	100 NO	2.23 ^b^	235/285	N/A	N/A N/A	[149]
Ag@TiO_2_ (NSs)	Hydrothermal	300	100 C_2_H_5_OH	8.5 ^a^	9/10	N/A	30 days and 5 cycles	[150]

^a^—R_a_/R_g_, ^b^—(R_g_-R_a_)R_a_.

## Data Availability

Data sharing not applicable.
